# RNA element discovery from germ cell to blastocyst

**DOI:** 10.1093/nar/gky1223

**Published:** 2018-12-21

**Authors:** Molly S Estill, Russ Hauser, Stephen A Krawetz

**Affiliations:** 1 Center for Molecular Medicine and Genetics, Wayne State University, Detroit, MI 48201, USA; 2 Vincent Memorial Obstetrics and Gynecology Service, Massachusetts General Hospital, Harvard Medical School, Boston, MA, USA; 3 Departments of Environmental Health and Epidemiology, Harvard T.H. Chan School of Public Health, Boston, MA 02115, USA; 4 Department of Obstetrics and Gynecology, Wayne State University, Detroit, MI 48201, USA

## Abstract

Recent studies have shown that tissue-specific transcriptomes contain multiple types of RNAs that are transcribed from intronic and intergenic sequences. The current study presents a tool for the discovery of transcribed, unannotated sequence elements from RNA-seq libraries. This RNA Element (RE) discovery algorithm (REDa) was applied to a spectrum of tissues and cells representing germline, embryonic, and somatic tissues and examined as a function of differentiation through the first set of cell divisions of human development. This highlighted extensive transcription throughout the genome, yielding previously unidentified human spermatogenic RNAs. Both exonic and novel X-chromosome REs were subject to robust meiotic sex chromosome inactivation, although an extensive de-repression occurred in the post-meiotic stages of spermatogenesis. Surprisingly, 2.4% of the 10,395 X chromosome exonic REs were present in mature sperm. Transcribed genomic repetitive sequences, including simple centromeric repeats, HERVE and HSAT1, were also shown to be associated with RE expression during spermatogenesis. These results suggest that pervasive intergenic repetitive sequence expression during human spermatogenesis may play a role in regulating chromatin dynamics. Repetitive REs switching repeat classes during differentiation upon fertilization and embryonic genome activation was evident.

## INTRODUCTION

Expression profiles of known RNAs have been catalogued for a range of cell types, with the use of expression arrays and, more recently though RNA deep-sequencing studies. This has yielded a series of useful databases including GTEx (https://www.gtexportal.org/home/), EMBL-EBI’s Expression Atlas (https://www.ebi.ac.uk/gxa/home/), The Human Protein Atlas (https://www.proteinatlas.org/), and ENCODE (www.encodeproject.org) (1–6). These databases and RNA-seq studies generally focus on annotated genes and transcript variants that are derived from transcript modeling programs such as Cufflinks (7) and are provided as part of the Refseq and Gencode annotations (8,9).

Both coding and non-coding RNAs play major roles in all cellular processes. In addition to protein-coding RNAs, at present, there are 48 different non-coding and pseudogene classes of RNA documented in the version 27 annotation of the Human Gencode. Approximately 40% of the annotated genes in Gencode correspond to long and short non-coding RNA genes (10). Non-coding intergenic regions are known to contain regulatory RNAs. These include long intergenic non-protein coding RNA (lincRNA), enhancer RNA (eRNA), piwi-interacting RNA (piRNA) and circular RNAs, with others just beginning to be described (11–14). The human transcriptome is likely to be more complex than even these annotations indicate, as an estimated three quarters of the human genome is transcribed (15). This would include novel tissue-specific RNAs, whose roles remain to be established (16).

The palette of RNAs appear enriched in certain specific tissues, with each providing a specialized function, e.g., brain—cognitive and functional system level control, and germline—stem cell—defining development (17–19). Their corresponding complexity is exemplified in the testis by the collection of unique structural and functional spermatozoal-specific transcript variants (20) that are observed during maturation, as sperm assume their unique shape. This culminates with the compaction of the sperm nucleus to a transcriptionally and translationally inert structure. The latter is ensured by fragmenting rRNAs (21), as well as others and completes with the expulsion of the majority of the cytoplasm. In addition to the paternal genome and sperm encapsulated RNAs (22), RNA/proteins and other molecules from distant tissues acquired during epidydimal transit (23,24) are delivered at fertilization. This provides a pathway for soma-to-germline transmission (22,25,26) that perhaps conveys signals echoing how other tissues have responded to the environment (reviewed in (27)).

We have previously shown that unannotated transcripts corresponding to intronic and intergenic regions of the spermatogenic genome are comparatively abundant in human sperm (20,28–30). They vary amongst species and in response to and can provide markers of disease (30–32). These observations drove the development of this algorithm to systematically identify the genomic locations of RNAs, defined as RNA elements (RE), i.e., regions transcribed throughout the genome. This unbiased analysis tool is not limited to those RNAs currently defined in the databases, as it does not seek to generate gene structures from REs. It is compatible with a range of Next Generation Sequencing (NGS) platforms, RNAs from varied sources, abundance, quality, and levels of fragmentation, i.e., FFPE-like RNAs. The algorithm only requires the BAM file of genomic alignments to detect transcribed regions of novel loci in conjunction with well-known annotated loci.

In the current study, the RE discovery algorithm was applied from the perspective of the human male germ cell to blastocyst paradigm. A series of spermatogenesis and embryogenesis pattern specific intergenic human REs were identified, indicating that the transcriptome extends well-beyond the annotated genes, including those delivered at fertilization. Tissue-specific REs comprised of intronic and intergenic REs were uncovered and, in some cases, exon boundaries extended. Transcribed genomic repetitive sequences, such as simple repeats, HERVE, and HSAT1, were shown to be associated with RE expression during spermatogenesis, and may play a developmental stage specific role. Similarly, in the human embryo, MER73 was associated with RE transcription at the minor wave of zygotic genome activation and MLT2A1 and SVA-D expressed through the major wave during the transition to the embryonic genome. Overall, this study provides a deeper understanding of the dynamic transcriptome of human sperm, as well as uncovering the possible role of specific repetitive sequences in the spermatogenesis.

## MATERIALS AND METHODS

### RE discovery

The current study used Gencode release 26 (for GRCh38) and the GRCh38 genome for RE discovery, which is detailed in Supplemental Appendix A. RNA-seq samples used in RE discovery are described in Supplementary Table S1. Sample reads were pre-processed prior to RE discovery with Trimmomatic version 0.36, trimming Illumina adaptors and poly(A^+^) sequences, where appropriate, with parameters ‘LEADING:3 TRAILING:3 SLIDINGWINDOW:4:15’. Reads were aligned to the GRCh38 genome using HISAT2 (version 2.0.6), using the parameters ‘-p10 -max-seeds 30 -k 2’. Read coverage was provided to the RE discovery tool in bigwig format, generated by converting BAM files to bedgraph format, using the bedtools tool genomeCoverageBed, with the parameters ‘-split –bg’, and subsequently bigwig format, using the bedGraphToBigWig program (available from the UCSC Genome Browser utilities). The threshold parameter μ for RE discovery was set to 2.5 reads per million, to minimize their contribution of background noise. Novel REs from each study were combined using custom R commands, which merged overlapping novel REs, re-annotated the merged REs, and added the merged REs to the exonic REs, to produce a collective set of REs. The collective set of REs for the different samples is given in Supplementary Table S2 and was subsequently used in all analyses. For comparison of RE discovery to established transcript-building software, Cufflinks (v2.2.1) and Stringtie (v1.3.4) were used on the same pre-processed reads previously used for RE discovery (7,33). Default parameters for both Cufflinks and Stringtie were employed, using Gencode release 26 (for GRCh38) as the reference annotation.

### Differential expression (LMEM, fold change, LM)

A linear mixed-effects model (LMEM) was used to calculate differential expression between poly(A^+^) and total RNA libraries from oocyte through early embryonic development (34–36). The LMEM was used with a random slope and intercept for each cell type, to consider heterogeneity across cell types [formula = RPKM ∼ RNA.type + (1 + RNA.type | Tissue.simple)]. Residuals of randomly selected REs were analyzed for homoscedasticity, ensuring that the assumptions of the LMEM were satisfied. Multiple testing correction was applied to *P*-values for resultant slopes, using Benjamini-Hochberg correction (37).

Differential expression of poly(A^+^) and total RNA libraries in sperm and testis tissue was determined using a fold-change (fold change $ = \ {\rm log}_2 \left( {\frac{{{\rm mean}( {{\rm Total}\ {\rm RNA}} )}}{{{\rm mean}( {{\rm poly}( {{{\rm A}^ + }} )} )}}} \right))$. The use of an expression ratio, rather than linear modeling, was necessary due to the technical differences between the total RNA sperm samples (30) and the three poly(A^+^) sperm libraries (38), as well as the absence of multiple independent total RNA testis samples (20).

### RE enrichment for repetitive sequences

In cases when median RE RPKM in spermatozoa exceeded 1 RPKM (thus removing REs with low coverage in most samples), peak RE RPKM was 25 RPKM and subsequently used as an expression threshold (Supplementary Figure S1B). REs were first assigned as ‘Expressed’ if the median RPKM for the cell/tissue type was >25 RPKM. The enrichment or depletion of repetitive sequences in the expressed REs was calculated using UCSC’s Repeatmasker track (for GRCh38), a hypergeometric test and custom R code. The proportion of each genomic repeat in all available REs was used as input probability, with the number of expressed REs for the given cell type used as the sample size. The probability of drawing the actual number of expressed REs overlapping the given repeat type was adjusted using a Bonferroni correction (39). To identify repeats of interest, significantly enriched or depleted repeats were additionally filtered to remove repeats with minimal over- or under-enrichment. Thus, only repeats whose difference between the expected and observed RE count was >10 REs were retained.

### Expression clustering

Expression patterns across spermatogenic cell types were identified using the R package Mfuzz (30,40–42). A total of 20 cluster patterns were generated per analysis, with a minimal membership value of 0.7 required to assign a RE to a given cluster.

### Paternal/maternal transmission

REs were assigned as maternally transmitted to the zygote with median zygotic level >10 RPKM, sperm <2 RPKM and oocyte >25 RPKM. REs were assigned as paternally transmitted with moderate confidence with median zygotic level >10 RPKM, sperm >25 RPKM and oocyte <5 RPKM. REs were assigned as paternally transmitted with greatest confidence with median zygotic level >10 RPKM, sperm >25 RPKM and oocyte <2 RPKM.

### FDR calculation for GTEx and PPV calculation for sperm

The accuracy of the RE discovery algorithm to identify expressed loci was calculated using the Jodar *et al.* dataset, which consisted of seven fertile human sperm samples, prepared using total RNA (30). RE discovery was performed on the seven samples, at a range of μ from 1 to 10 RPM, at increments of 0.5 RPM. The RPKM of the resulting novel REs for each sample was calculated, along with the median RPKM across the seven samples. Experimental thresholds for calling a RE as ‘expressed’ ranged from 1 to 200 RPKM, at increments of 1 RPKM. At each expression threshold, the number of REs with a median RPKM at or exceeding the threshold were recorded. The positive predictive value (PPV) at each expression threshold and μ was calculated as

\begin{eqnarray*}{\rm PPV} = \frac{{{\rm Novel}\ {\rm REs} >{\rm Expression}\ {\rm threshold}}}{{{\rm Novel}\ {\rm REs} > {\rm Expression}\ {\rm threshold} + {\rm Novel}\ {\rm REs} \le {\rm Expression}\ {\rm Threshold}}}\ \end{eqnarray*}

The ability of the RE approach to recapitulate tissue expression in the established databases was determined using the testis expression in the GTEx database (3). The median TPM for all GTEx testis samples was downloaded and was processed to replace duplicated common gene names with the mean TPM for all instances of the gene name. Only gene names found in both GTEx and exonic REs were used in subsequent intersect analysis. The unique gene names with expression exceeding 5 TPM were compared to those of the exonic REs exceeding 25 RPKM.

### Gene ontology

Ontological analysis was performed with the Genomatix software suite (https://www.genomatix.de/), version 3.10. The GeneRanker function (using Genomatix Eldorado version 12-2017) generated the ontological enrichment of signaling pathways.

## RESULTS

### RE identification and classification

**Figure 1.**
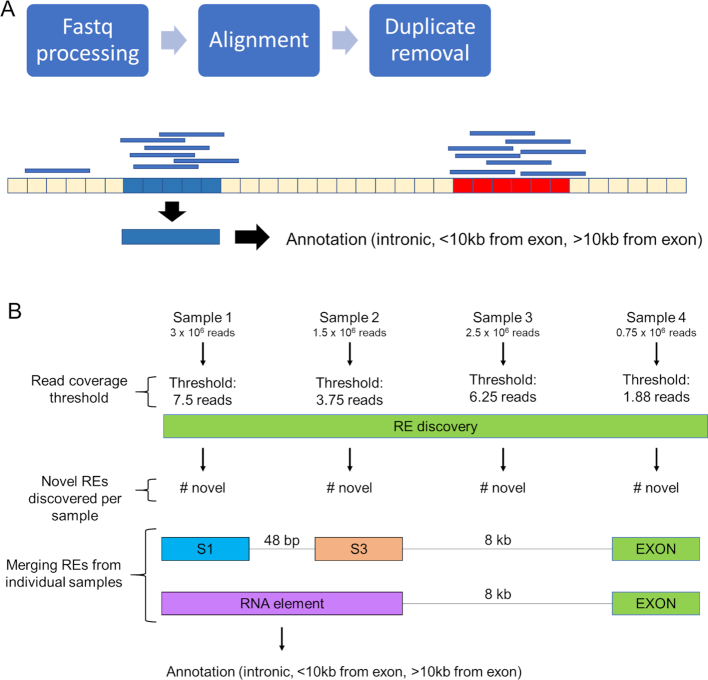
Pre-processing for RE discovery. (**A**) RNA-seq reads, in fastq format are processed to remove low-quality bases and adaptor sequences. The trimmed reads are then aligned to the genome, and the duplicate alignments removed. Read coverage is then used to identify 10 bp segments with read coverage surpassing μ. The expressed 10 bp segments (shown in blue) are merged and annotated according to their adjacency to exons (shown in red). (**B**) RE discovery workflow for theoretical RNA-seq samples. Each sample has a different library size, and correspondingly, different read coverage thresholds at a μ of 2.5 reads per million (RPM). Non-exonic regions of read coverage surpassing the assigned threshold are deemed ‘Novel REs’. Merging novel REs from the four different samples, yields two novel REs, one from Sample 1 (S1) and one from Sample 3 (S3) that are separated by up to 150 bp. Novel REs of the different samples S1 and S3 are merged into a final RNA element, represented in purple. Exonic REs are excluded from this merging step. The final novel RE set for the four samples is then annotated as Intronic, Near-Exon, purple, (<10 kb from exon), and Orphan REs (>10 kb from exon).

The RE discovery algorithm was designed to detect expressed regions of the genome using RNA-seq, regardless of the sequencing platform or read structure. A detailed description of RE discovery is presented in Supplementary Appendix A, and the corresponding code is provided online (https://github.com/mestill7/RE_discovery). Briefly, the known gene annotation (e.g. RefSeq, Ensembl, Gencode) for the genome of interest is parsed into individual exon locations. In the current study, Gencode release 26 (GRCh38) was used, with non-coding entries considered as annotated ‘exons’ (10). As summarized in Figure 1A, RE discovery first requires the sequenced reads to be processed, e.g. adaptors trimmed and low-quality bases removed, prior to alignment to the genome of interest. For the unannotated regions of the genome, the mean read coverage was calculated for each 10 bp genomic segment and the 10 bp segments with sufficient read coverage, determined by a threshold μ, retained. For the purposes of this study, μ = 2.5 reads per million provided well-balanced signal to noise ratio (Supplemental Supplementary Figure S2) that was suited for RNA libraries generated from low-input, potentially fragmented RNAs, as is often found in clinical formalin-fixed paraffin-embedded (FFPE) samples and spermatozoa (20,43). The overlapping 10 bp regions were subsequently merged to yield the final novel REs for each collection of samples studied. The merging steps allow for a maximum of 150 bp between element bins, intended to allow for gaps in coverage caused by sequencing bias and/or biological fragmentation.

**Figure 2.**
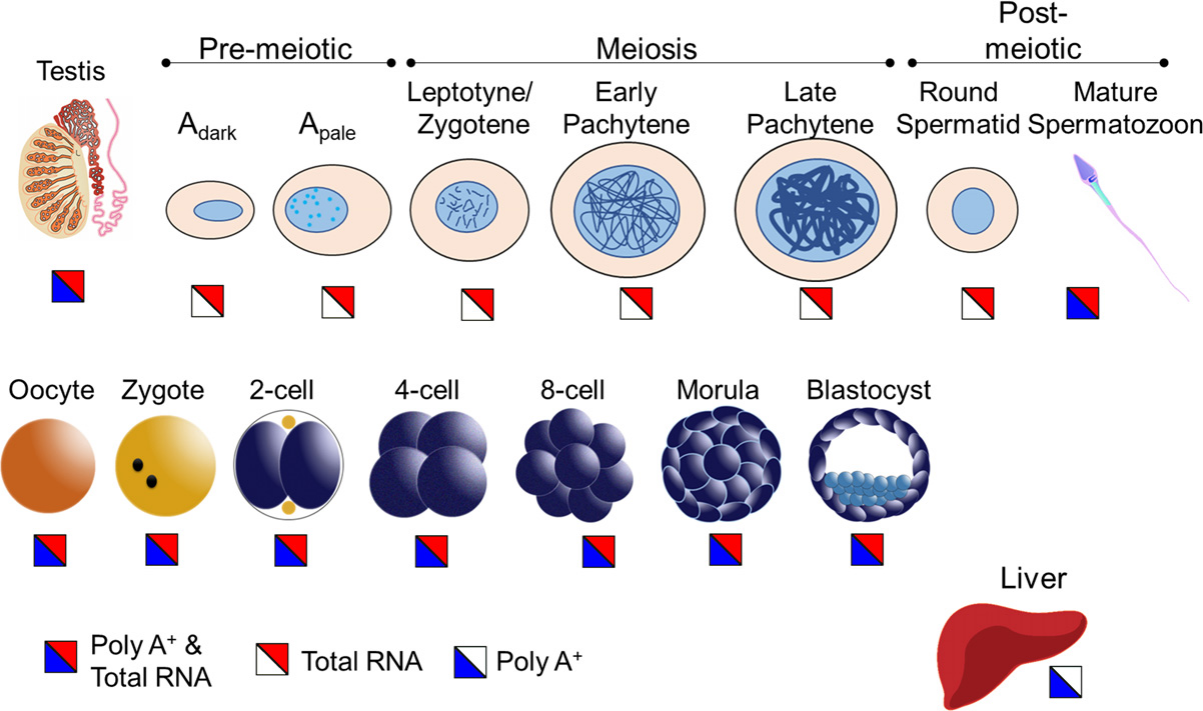
Tissue types used for RE discovery. The male germline within the testis tissue is divided into and represented by seven stages of spermatogenesis. The female germline is represented by a single-cell oocyte with embryonic stages that range from zygote to blastocyst. Somatic tissue is represented by the liver sample. Total RNA or poly(A^+^) enriched RNA-seq libraries are indicated in split squares, with blue representing poly(A^+^) selected samples, red indicating total RNA samples, and a split blue/red square as both library preparations.

Novel REs were then annotated according to their genomic position, relative to known exons (Figure 1B). ‘Intronic’ REs were located within introns, while any non-intronic REs located within 10 kb of an annotated exon were designated ‘Near Exon’ REs; ‘Orphan’ REs were at a distance greater than 10 kb from any known exon. An exonic RE was extended into a near exon RE if they were within 50 bp and the difference in read coverage was <50%. As summarized in Figure 2, previously published RNA-seq studies representative of human spermatogenesis, mature sperm, oocyte, embryonic stages, and liver samples, detailed in Supplementary Table S1, were subject to RE discovery. This set of RNA-seq libraries encompassed both poly(A^+^) selected and total RNA preparations. A database of REs across the different tissue and types was created by merging the novel REs from each study with the exonic and non-coding transcript REs (Supplementary Table S2) and used in all subsequent analyses. For any given tissue type, the majority of REs are lowly expressed, necessitating filtering of lowly expressed REs prior to analysis (Supplementary Figure S1A and B). RE length was on average higher in exonic REs compared to novel REs (Supplementary Figure S1C).

The above RE identification method was developed to ensure accuracy in face of extensive RNA fragmentation, naturally occurring in human sperm. Certain tissue preparations, such as FFPE, also yield compromised RNA preparations. Given that several established transcript-building algorithms are readily available, we compared both Stringtie (v1.3.4) and Cufflinks (v2.2.1) to the RE approach for two random sperm samples and two male human cell lines. RNA-seq datasets from human cell lines, i.e. SRR020288 (h1 hESC) and SRR3192556 (OCI-LY7, derived from a B cell lymphoma), provided independent datasets when testing the RE method. Using minimal thresholds of expression (>10 RPKM in REs, >1 FPKM in Cufflinks and Stringtie), the majority of expressed REs overlap locations of transcripts generated using transcript-building software. Across the four samples, 67–92% of ‘expressed’ REs overlap Stringtie results, and 81–90% overlap Cufflinks results at the above thresholds of expression (Supplementary Table S3). Notably, regardless of the transcript-building method and required expression thresholds, a majority of REs (complete range 21–93%) lacking overlaps with Cufflinks and Stringtie results are Exonic REs, suggesting that the established transcript-building methods are less than ideal for fragmented or unevenly covered transcripts.

With the function of the novel REs being unknown, we hypothesized that the novel REs may have regulatory roles. To assess this, REs were overlapped with a series of epigenetic marks and regulatory genomic sequences (Supplementary Figure S3). For regulatory chromatin marks (proximity to DNase hypersensitive regions, proximity to CTCF binding sites, proximity to topologically associating domains (TADs), and proximity to ENCODE Transcription Factor Binding Sites (TFBS)) (44–49), the novel RE classes largely showed a similar overlap proportion as Exonic REs. All RE classes showed very little overlap with piRNA clusters (50,51). Notably, all classes of novel REs had a high overlap (>50%) with repetitive sequences (UCSC’s Repeatmasker track (for GRCh38) (52), compared to the ∼22% overlap in Exonic REs.

### Poly(A^+^) selection reduces RNA-seq complexity

**Figure 3.**
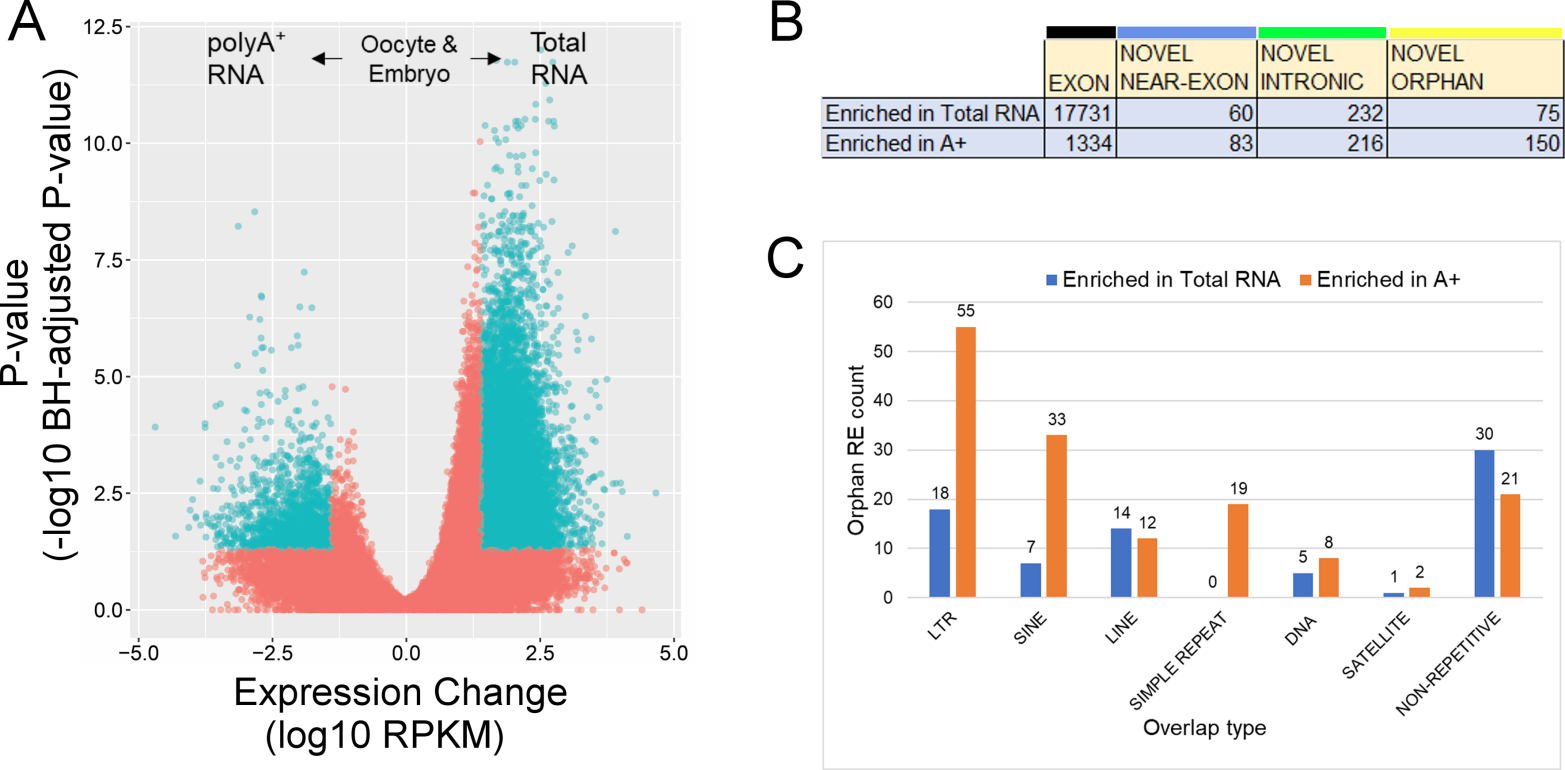
Orphan REs are enriched in poly(A+) samples. (**A**) Volcano plot of slope changes in REs from LMEM in oocyte and embryo, with the X-axis representing slope change in log_10_-transformed RPKM, and the Y-axis representing the Benjamini-Hochberg-adjusted *P*-value as a negative log10-transformed *P*-value. Positive slope and negative slope indicate increased abundance in total RNA and poly(A^+^) preparations, respectively. Each point represents a single RE, with blue points indicating statistically significant REs (adjusted *P*-value < 0.05) with absolute slope changes exceeding 25 RPKM. (**B**) Distribution of REs enriched in either total RNA or poly(A^+^) libraries, according to the annotation class. (**C**) The distribution of Orphan REs enriched in either total RNA or poly(A^+^) libraries, according to repeat class.

The RE discovery algorithm was developed to identify transcribed intergenic loci from RNA-seq data. Many novel loci (e.g. Near-exon and Orphan REs) were hypothesized to be derived from non-polyadenylated RNAs, since this class appears underrepresented in the genome and the GENCODE annotations. A series of poly(A^+^) selected and Total RNAs from a range of cell types that capture the period from fertilization to early embryonic development from oocyte, zygote, 2-cell embryo, 4-cell embryo, 8-cell embryo and morula (Figure 2) and the male germline, through ejaculated sperm and testis, were examined (34–36). Applying a linear mixed-effects model (LMEM) to Total and poly(A^+^)-selected RNAs from the human oocyte and various stages of early embryonic development, revealed a comparatively lower number of REs detected within the poly(A^+^) selected fraction (Figure 3A). In general, the number of novel REs that were either increased or depleted by poly(A^+^)-enrichment do not markedly differ (Figure 3B). Interestingly, the number of Orphan REs approximately doubled upon poly(A^+^)-enrichment as compared to the Total RNA fractionation. This suggests that a population of Orphan REs belong to a larger, yet unknown set of polyadenylated transcripts. To determine if poly(A^+^) enrichment of Orphan REs reflected a specific class of genomic repeat, the distribution of repeats within the 150 poly(A^+^) enriched Orphan REs was assessed and is shown in Figure 3C. Within the 129 Orphan REs that contain a repetitive element, the majority are LTRs and SINEs. It is worth noting that 40 of the 55 LTR-containing REs are ERVL-MaLRs (Supplementary Table S4). This is a non-autonomous LTR-retrotransposon element derived from ERV (53,54) that may function in regulating gene expression during the oocyte-to-embryo transition (55).

The effect of poly(A^+^) enrichment was also assessed individually for human sperm and testis samples, providing the other half of the equation to early post-fertilization development. Poly(A^+^) enrichment has contrasting effects on exonic REs in spermatozoa and testis, with poly(A^+^) enrichment depleting and enriching exonic REs in sperm and testis, respectively. However, unlike embryos, novel REs are markedly enriched in sperm and testis Total RNA libraries, reflective of the relatively uncharacterized state of this cell type (Supplementary Figure S4). Although poly(A^+^) enrichment does effectively reduce RNA library complexity, it does not appear to select for RNAs of any given biological function or pathway, with many GO terms shared in both the poly(A^+^)-enriched and Total RNA-enriched gene sets of the human embryo (Supplementary Table S5).

### Round spermatids and mature sperm have numerous intergenic SREs

**Figure 4.**
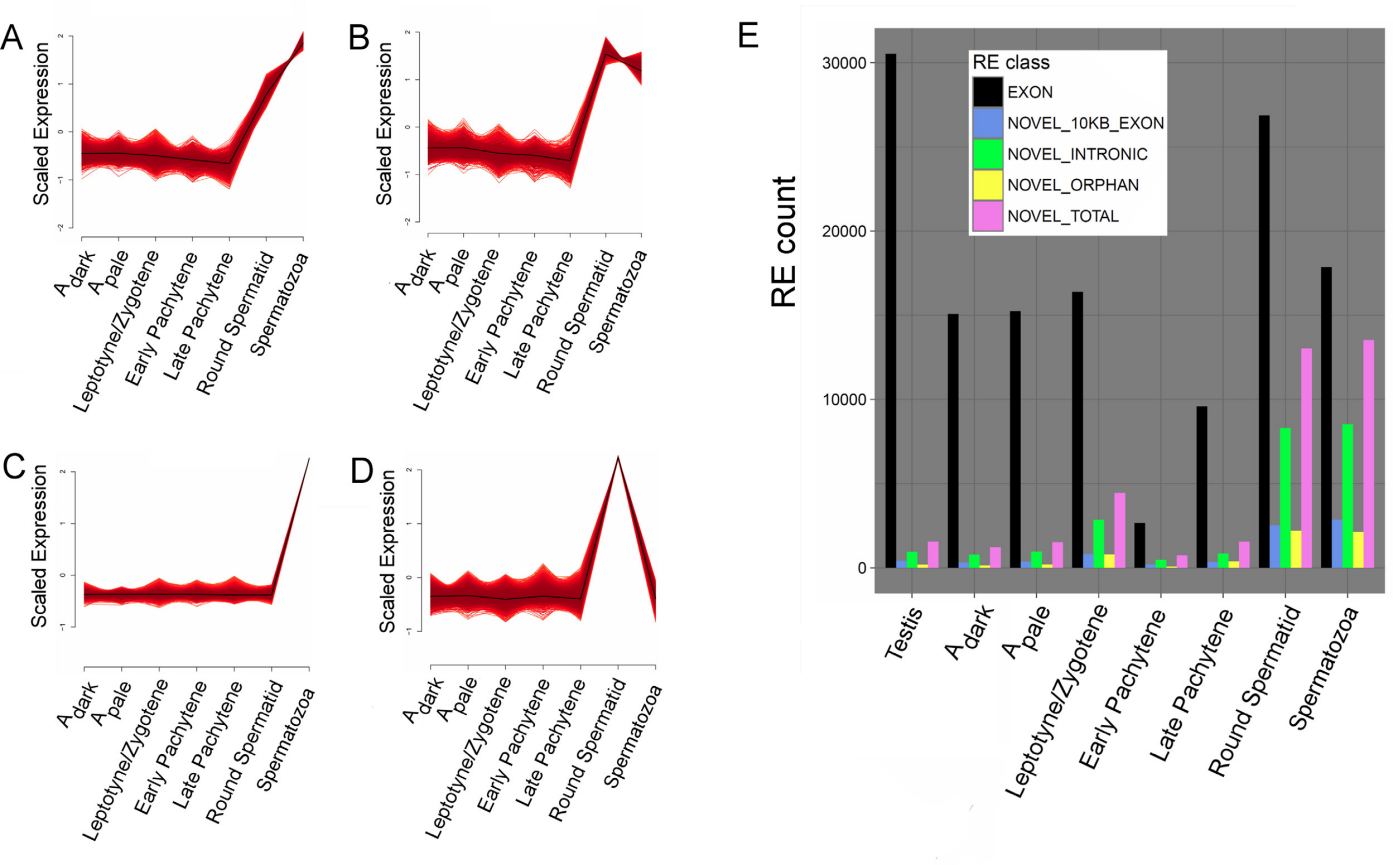
Mfuzz clusters highlighting the round spermatid to spermatozoon transition. (**A**–**D**) Clusters with increased expression in round spermatids and/or mature spermatozoa. (**E**) RNA element abundance as a function of annotation class and cell type with median RPKM >25.

RE expression throughout spermatogenesis was examined as a comparison to previously published patterns of whole transcript expression during the spermatogenic cycle(42). The spermatogenic stages encompassed six cell types (Spermatogonia through Round Spermatids), isolated using laser capture microdissection (42). Clustering of the various REs expression patterns across spermatogenesis was initially performed using Mfuzz, (40,41) with the published six cell types (42). As shown in Supplementary Figure S5, RE expression across spermatogenesis recapitulated those patterns previously observed using whole transcripts (values for each RE are provided in Supplementary Table S6). To extend the analysis to the final stage of spermiogenesis, RNA-seq from ejaculated sperm datasets from fertile males (30) were included (Figure 2). The addition of mature sperm enabled the discovery of several patterns specific to early round spermatids and maturing round spermatids, as observed through mature spermatozoa (Figure 4A–D). The final stages of spermatogenesis involve a burst of transcription, as well as the formation (and eventual loss) of the residual body as the majority of the cytoplasm is expunged from the cell. The burst of transcription in round spermatids is observed as a general increase in transcription of exonic REs that include 34,226 REs found in round spermatids but not in the late pachytene stage spermatocytes. Interestingly, a large portion of spermatid and/or mature sperm-specific clusters are generated from novel REs, suggesting that intergenic and intronic REs play a substantial role in the final stages of spermatogenesis that forms each spermatozoon as summarized in Supplementary Table S7. To verify these observations, expressed (median expression >25 RPKM) REs for each spermatogenic stage were partitioned according to RE class (Figure 4E). The vast majority of REs expressed in pre-meiotic and meiotic stages were exonic (85 ± 7%). This was followed by a notable increase in the number of novel REs in Round Spermatids and Spermatozoa. The contribution of novel REs to the total transcriptome rose to 47% in mature sperm.

Ontological analysis of the exonic and novel REs (with the exception of Orphan REs) showed that the most abundant REs in round spermatids were enriched for genes involved in organelle biogenesis and maintenance. This is in accord with the physiological changes occurring during spermiogenesis. REs that are abundant in both round spermatids and spermatozoa were enriched for TNF-alpha signaling, associated with maintaining a homeostatic state (56,57). REs that were primarily abundant in spermatozoa were associated with a range of signaling pathways, such as Glutamate Receptor signaling, WNT signaling, NGF signaling, EGFR1, and Signaling by Rho GTPases (Supplementary Table S8). WNT signaling has several roles in spermatogenesis, from maintenance to maturation, and thus motility (58–60). The role of NGF signaling in spermatogenesis in humans is unclear but has been implicated in mammalian Sertoli-germ cell signaling, sperm motility, and the acrosome reaction (61,62). Sperm EGFR activation is a major driver of sperm capacitation (63,64), while Rho GTPases are likely to aid as mediators of the acrosome reaction (65). Odorant receptors may be required for sperm chemotaxis in mammals (38), while glutamate receptors may also be involved in capacitation and/or sperm chemotaxis (66,67) although such functions have yet to be demonstrated in mammalian systems.

### Sex-chromosome expression during spermatogenesis

Meiotic sex chromosome inactivation (MSCI), the process by which genes located on the X-chromosome are repressed during meiosis, is essential for successful meiosis during human spermatogenesis (68). However, abundant evidence suggests that numerous X-linked genes escape post-meiotic X chromosome silencing (PMCI), a process that may be less effective in humans than other species (69,70). In comparison, most classes of Y-linked REs undergo silencing during MSCI, with the exception of Y-linked Orphan REs that are present throughout spermatogenesis.

**Figure 5.**
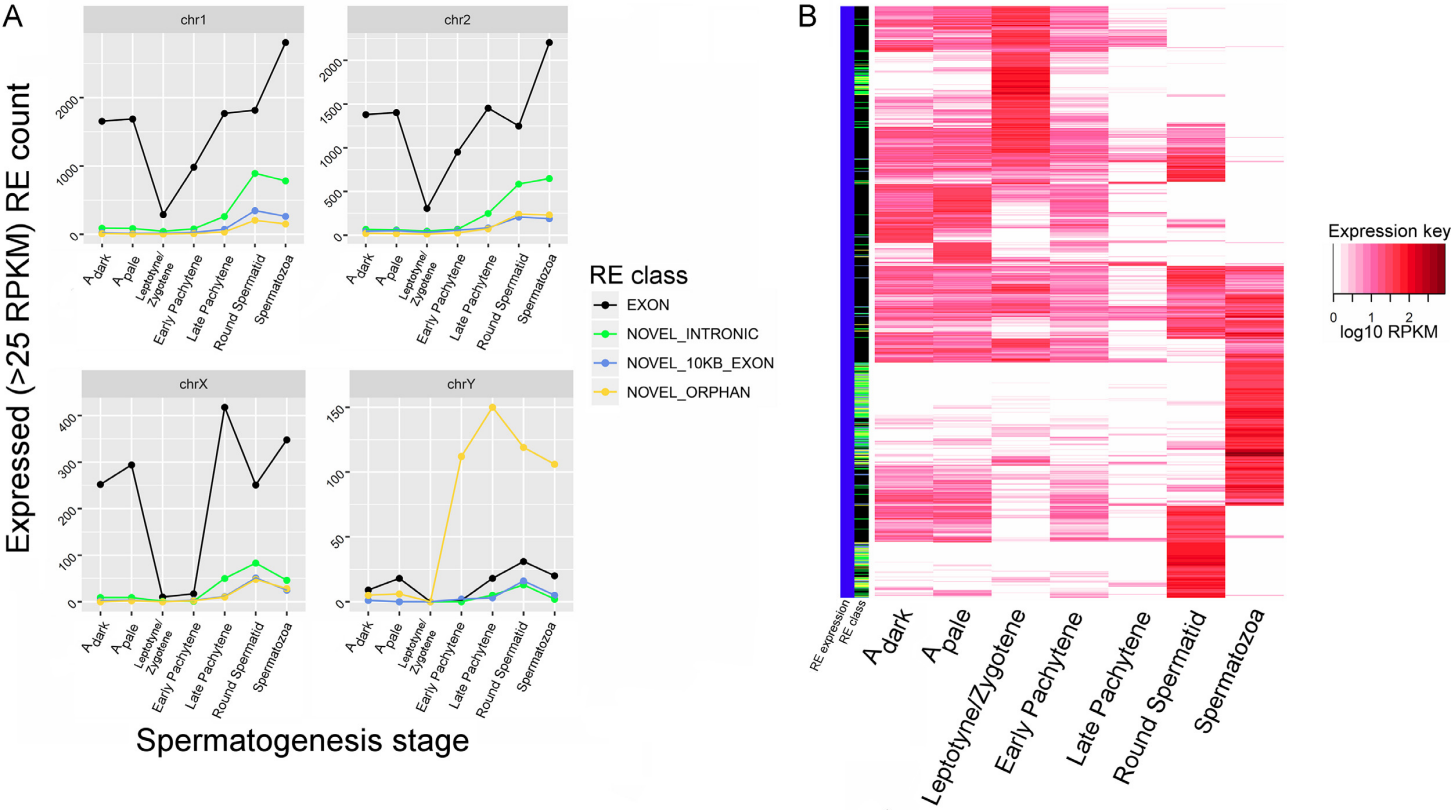
X-chromosome expression during spermatogenesis. (**A**) The number of expressed REs across each spermatogenic stage, for two representative autosomes (upper panels chr1 and chr2) and the sex chromosomes (lower panels chrX and chrY). The connected points are colored according to the RE class, with Exonic REs in orange, Intronic REs in green, Near-exon REs in light blue, and Orphan REs in purple. The X-axis of each graph presents the spermatogenic stage, with the pre-meiotic stages represented by A_dark_ and A_pale_, the meiotic stages represented by Leptotene/Zygotene, and early/late Pachytene, and the post-meiotic stages represented by round and mature sperm. (**B**) An expression heatmap of X-chromosome REs that are primarily expressed (>25 RPKM) at one spermatogenic stage. RE class, shown adjacent to the RE expression column, shows Exonic REs in black, Intronic REs in green, Near-exon REs in light blue, and Orphan REs in yellow.

As shown in Figure 5, repression of exonic X-linked REs during spermatogenic MSCI is evident. This is followed by de-repression of X-linked exonic and novel REs, that return to pre-meiotic levels in mature sperm (Figure 5A). Notably, several X-linked REs are intensely expressed (at a threshold of 25 RPKM) in solely one spermatogenic stage, including the post-meiotic stages, i.e., round spermatids and to a greater extent, mature sperm (Figure 5B). Overall, the patterns of X-linked REs across spermatogenesis imply a larger upregulation of genes and novel REs in the post-meiotic stages than previously thought, with the number of expressed X-linked REs largely following the patterns laid by autosomes. We note that two of the 289 paternally transmitted REs were located on the X-chromosome, and both were exonic REs. The two REs are located (in hg38 coordinates) at chrX_2717605_2717652 and chrX_149929645_149930127, corresponding to CD99 and XX-FW81066F1.2, respectively. The spermatogenic roles of CD99, a cell surface glycoprotein involved in T-cell adhesion processes, and XX-FW81066F1.2, a poorly described transcript with a putative protein structure or antisense lncRNA function (5), are unknown.

### Paternal transmission of REs

It has been proposed and shown *in vitro* that human sperm deliver a cadre of RNAs upon fertilization (20,27,71,72). In the current study, the series of human RNA-seq profiles from sperm, oocyte, and embryo allowed for the identification of REs that are transmitted to the human oocyte solely by sperm. These are in addition to those 26,740 zygotic REs (5% FDR), associated with a total of 6,118 individual named genes, which are essentially provided by the oocyte, but not present in sperm (Supplementary Figure S6). Up to 289 sperm REs were identified as a majority contributed by paternal transmittance, with an FDR of ∼3.4%, and 75 REs essentially provided by the sperm, at an FDR of ∼2.7% (Supplementary Figure S7A,B and Table S9). Interestingly, the 289 REs were enriched for ‘cycling of RAN in nucleocytoplasmic transport’ (*P* = 8.36 × 10^–8^) and the Unc 51 Like Kinase (*P* = 1.47 × 10^–3^). RAN cycling is required for effective translocation of RNA and proteins across the nuclear pore. The human sperm REs contain RANGAP1, XPO7, XPO6, NUP210 and NUP214, as members of the nucleoporin complex. Interestingly others have shown that at least in embryonic stem cells, the nucleoporin complex may regulate parentally imprinted genes (73). In comparison, the Unc 51 Like kinase is associated with autophagy a process that is essential for the oocyte-to-embryo transition (74). These observations are consistent with the view that the paternal RNAs may contribute to re-establishing nuclear transport in the zygote and clearance of extraneous cellular complexes post-fertilization, when cell lineages begin to be established.

### Differential gene expression during embryogenesis

Transcriptomic changes across mammalian embryogenesis have been well-studied, using both microarrays and RNA-seq (75–79). However, these experiments have not addressed the contribution of intergenic RNAs to embryogenesis and, importantly, during human embryogenesis. Towards filling this gap, we examined expression changes of novel REs from oocyte to blastocyst while considering the contribution of the spermatozoon, testing the hypothesis that both exonic and novel REs would exhibit distinct patterns.

**Figure 6.**
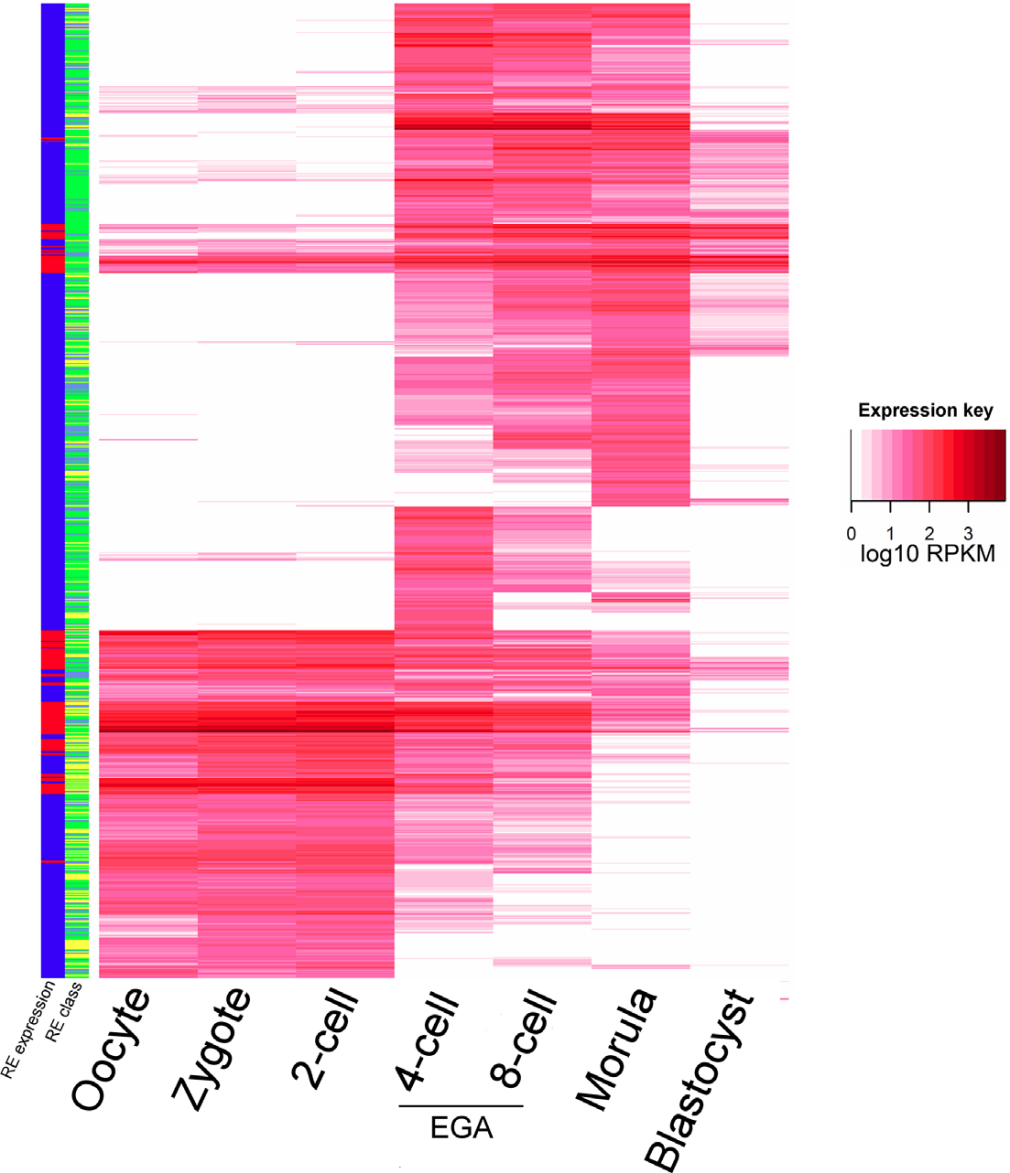
Differential novel REs across embryogenesis. The overall expression level is represented as left panel ‘RE expression’, with red indicating a median expression exceeding 25 RPKM. RE class, shown adjacent to the RE expression column, shows Intronic REs in green, Near-exon REs in light blue, and Orphan REs in yellow. The REs presented from oocyte to blastocyst are differentially expressed across at least one developmental stage.

To identify differentially expressed REs, a linear model was applied to the single-cell oocyte and embryonic RNA-seq datasets (35,80). Differential expression with REs reiterated previous analysis of RefSeq-annotated genes suggesting that the oocyte, zygote, and 2-cell embryo contain a similar distribution of transcripts (35). Few differences (59 REs) were identified between oocyte and zygote, and no differential REs were identified between zygote and 2-cell embryo (Supplementary Figure S8). As expected, exonic REs exhibited characteristics of maternal genes, which are supplied by the oocyte and diluted as the embryo develops in anticipation of the 4- and 8-cell stage extensive Embryonic Genome Activation (Supplementary Figure S9) (81). This included a set of novel maternal REs specific to the early zygote (maternal genes) and EGA (the 4- and 8-cell stage). The majority of these novel maternal REs are intronic, suggesting e.g. (1) incomplete processing, (2) expression within an intron, (3) retention of circular RNA, or some other form. They are supplemented by a series of maternal intergenic Orphan REs. Interestingly, these REs also followed similar patterns, defining clusters of REs that are present during the minor first wave of human ZGA, as well as clusters that are active during EGA (Figure 6). While the novel REs with a maternal gene pattern are enriched for neuronal genes (Neuronal system, *P* = 2.12e–05), those expressed during EGA are associated with protein metabolism (*P* = 4.90e–06), consistent with the energy and synthesis requirements of the early embryo.

### Expression of repeats during spermatogenesis and early embryonic development

**Figure 7.**
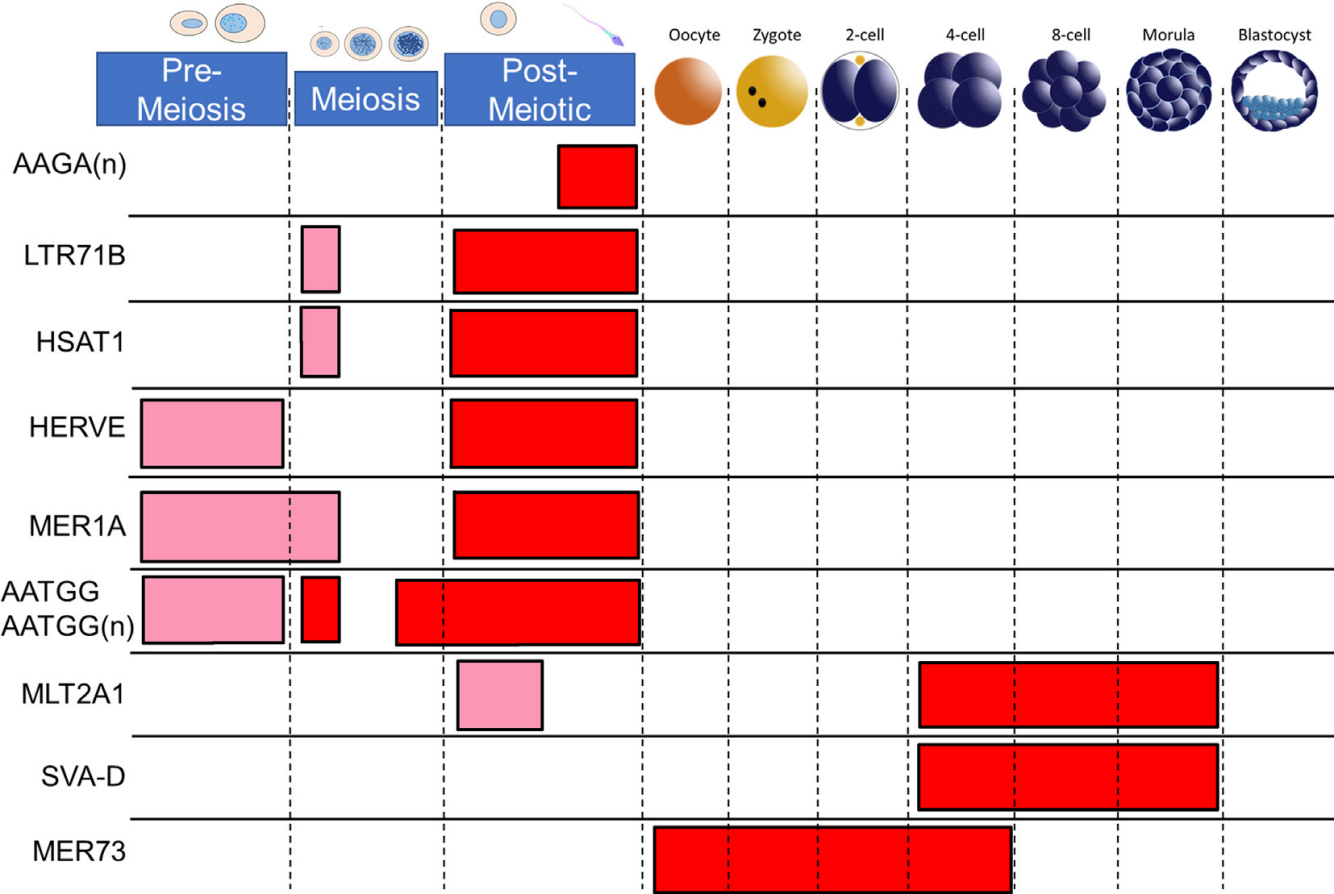
Expression of repetitive sequences across spermatogenesis and embryogenesis. Moderate enrichment (mean RE expression > mean expression across all cell types) is shown in pink, and strong enrichment (mean RE expression is an upper outlier) is shown in red. The name of the genomic repeat is given on the left of the diagram, and the cell type is shown at the top of the diagram.

Genomic repetitive elements and small non-coding RNAs are thought to play a role in confrontation-consolidation of the maternal and paternal genomes after fertilization (29,82). As novel REs tend to overlap genomic repetitive sequences, we employed RE expression to determine what genomic repeats may influence RNA expression throughout spermatogenesis and early human embryo development (Figure 7). The relative enrichment or depletion of repetitive sequences in the expressed REs was calculated for each available cell type. Briefly, the number of instances of genomic repeats overlapping expressed REs in each cell type were compared to an expected random distribution, with the random distribution drawn from the repeat occurrence in all available REs. Using a hypergeometric test, both relative enrichment and depletion of repeat families were calculated across cell types. Despite the many instances of repeat depletion, there were relatively few instances of enrichment.

Although several studies have examined the influence of environment on epigenetic marks, such as DNA methylation, at genomic repeats in spermatozoa, much less is known about genomic repeat expression during spermatogenesis and if genomic repeats are in part driving spermatogenesis, perhaps through transcriptional regulation or chromosomal reorganization (83,84). Four repeat families, LTR71B, HERVE-int, HSAT1 and MER1A were primarily expressed in both round spermatids and mature spermatozoa, while the centromeric repeat AATGG(n) showed greatest expression in the leptotyne/zygotene and late pachytene stages through the post-meiotic phase (85). The simple repeat AAGA(n) was enriched solely in mature spermatozoa. The genomic repeats identified here as expressed during spermatogenesis suggest that different repeats have different roles in spermatogenesis. For example, the centromeric repeat AATGG(n) likely plays a role in establishing stage specific chromosomal structure and position throughout spermatogenesis (86,87). The simple repeat AAGA(n) and HSAT1, primate-specific Satellite repetitive element, may also play a role in organizing sperm nuclear structure through Matrix-Associated Regions (MARs) of sperm, which are enriched in TTCT(n) and TCTT(n) repeats (87). The remaining spermatogenesis-associated repeats LTR71B, HERVE, MER1A are all members of the HERV family of retroviruses or DNA transposons. The murine embryo and sperm are known to express a LINE-1-encoded Reverse Transcriptase (RT) that may serve to reverse transcribe the sperm-supplied retroviral and transposon RNAs for integration into the genome (88–90). However, we note that the presence of LINE-1-encoded RT in mature murine spermatozoon, does not appear to be extended to an enrichment of LINE1 RNAs in human sperm. This likely reflects a species differences, although one cannot exclude the influence of differing methodologies. However, MLT2A1 and SVA-D are both present during EGA, while MER73 was strongly enriched in oocyte and the early embryo (Figure 7). Both MLT2A1 (primate-specific) and MER73 are LTRs for ERVL endogenous retrovirus, while SVA-D is a hominid-specific composite retroelement (SINE-R + VNTR + Alu) (54). Although SVA-D is a marker of naive human ESCs, consistent with the enrichment from 4 cell to morula stage, it is not enriched in blastocyst stage, from which human ESC cell lines are derived (91). The ERVL retrotransposon has been previously implicated in mammalian embryonic development (55).

## DISCUSSION

In this study, we sought to enrich our understanding of the transcriptome across the human germ cell and early embryogenesis. To accomplish this, we interrogated publicly available RNA-seq datasets using a new method ‘REDa’ to identify novel RNA elements (REs). This method was used to detect REs in differentiating cells of the germline, embryonic cells, and somatic tissues. The RE discovery algorithm possesses a robust positive predictive value (PPV) (Supplementary Figure S2), eliminating background signal even at the lower thresholds. Novel REs are annotated as Intronic, near-exon (within 10 kb of an exon), and Orphan (>10 kb from an exon), and are considered along with the previously known exonic REs.

The accuracy of the RE approach, which separates exons into individual units, rather than linking exons into a whole transcript, was tested by comparing expressed REs in testis libraries to the testis expression levels given by GTEx. At least 91% of gene names associated with testis-expressed exonic REs overlap with gene names expressed in GTEx testis tissue, suggesting that the RE approach can recapitulate the patterns designated in established expression databases. The accuracy of the RE approach was further tested on poly(A+) selected libraries, reiterating previous studies that indicate a reduction of transcript diversity and exon expression upon poly(A+) enrichment (92). The number of human zygotic LTR and SINE-associated REs that may be derived from poly(A+) intergenic transcripts is of note. In accord with the data of others (93–96), this could afford transcript stabilization and nuclear export (97) perhaps increasing their retention in a given cell of the dividing embryo. Notably, at least in mouse, the transcription of retrotransposon-derived RNAs is thought to impact chromatin accessibility, and thus embryonic development (98).

Isoform discovery approaches, such as Cufflinks and Stringtie (7,33), provide methods to suggest novel genes/isoforms, often relying on key structures like exon–intron junctions (99). Spermatozoal RNAs are often fragmented, limiting the efficacy of established transcript-building and differential expression algorithms. The RE method is solely intended for identifying expressed regions, which can subsequently be interrogated for the presence of novel isoforms or gene structures. A comparison of the RE approach to that of Cufflinks and Stringtie suggested that the established transcript-building methods are not sufficient for fragmented or unevenly covered transcripts. Additionally, the presence of spliced reads, a critical component to transcript-building, is reduced in spermatozoal RNAs (36% - 40%) compared to RNAs from cell lines (41–64%). We note that others have also employed a targeted Cufflinks (35) discovery approach to identify novel linear embryo transcripts. Reflective of the low level of expression and rigor required for identification, the majority of these linear transcripts were not discovered using the RE strategy (data not shown).

The transcriptome of the human male germline has largely been limited to the whole testis, with a few studies generating information from isolated germ cell populations from this mildly heterogeneous tissue. This contrasts with the mature mammalian spermatozoon, which is known to contain a complex transcript population and can be obtained in a relatively pure form (30,100). As described above, a large proportion of novel REs contribute to the post-meiotic phase of human spermatogenesis. GO analysis suggested that a range of signaling pathways, such as Glutamate Receptor signaling, WNT signaling, NGF signaling, EGFR1, and Signaling by Rho GTPases, are associated with REs present in ejaculated spermatozoa, with several of these pathways linked to sperm capacitation and the acrosome reaction. The TNF-alpha signaling associated REs enriched throughout the post-meiotic phase of spermatogenesis may be another part of a surveillance mechanism to ensure an optimal contribution (32).

Relatively few paternal full-length RNAs are likely to be exclusively contributed to the embryo (22). Of note, the genes associated with the paternally transmitted REs did not overlap those long RNAs suggested to be paternally derived in mouse (101). This is likely due to the differences in genome activation, which occurs in the late 1- cell zygote in mouse (102), compared to the later 4–8 cell stage of human embryos, or other sperm derived RNAs providing a substitutive function (20,103). The paternally transmitted REs in human were associated with RAN cycling and autophagy, suggesting that the paternal RNAs may contribute to re-establishing nuclear transport in the zygote and clearance of extraneous cellular complexes post-fertilization. Several paternal RNAs, all of which are expressed in human sperm (104), are generated from genes involved in RAN cycling (RANGAP1, XPO7, XPO6) or nucleoporins (NUP210, NUP214).

Although few paternally derived zygotic RNAs are X-linked, the expression patterns of REs located on the X chromosome are congruent with the current paradigm of Meiotic sex chromosome inactivation (MSCI) and reactivation during spermatogenesis (70). The current study also showed robust repression of exonic X-linked REs during spermatogenesis, as required for successful meiosis. A robust post-meiotic de-repression of exonic and novel X-linked exonic and novel REs also became apparent. The data suggest that the process of post-meiotic X chromosome silencing (PMCI) during human spermatogenesis is selective, as many genes and novel REs escape silencing.

The mechanism(s) driving spermatogenesis may involve the use of repetitive sequences as regulators of transcription and/or chromatin states (98,105,106). Its nuclear architecture reflects the complex and orchestrated compaction and restructuring of its chromatin via protamination. This is linked through the nuclear matrix/lamina in a non-random manner (107), consistent with the current 3D models (108). The enrichment of centromeric AATGG(n) repeat RNAs appears in the leptotyne/zygotene and late pachytene stages through the post-meiotic phase (85). This repeat can form a double folded hairpin (85), that in mice can promote RNA:DNA hybrids mediating heterochromatin formation (109). Perhaps this aids in excluding large repetitive DNA domains from homology searching enhancing the fidelity of meiosis as observed by the clustering of pericentromeric chromatin during meiosis (110).

A similar simple nuclear matrix/lamina associated repeat AAGA(*n*) that resides within the inner nuclear compartment (22) was enriched solely in mature spermatozoa yet does not appear in the oocyte or the developing embryo. As shown above four repeat families (LTR71B, HERVE-int, HSAT1 and MER1A) are transcribed coincident with the physiological changes of spermiogenesis with marked enrichment in both round spermatids and mature spermatozoon. LINE1 RNAs, which encode reverse transcriptase, were not enriched in human sperm or the zygote. However, the presence of an RT in the early embryo would provide the opportunity for LTR71B, HERVE-int and MER1A, components of HERVs and DNA transposons, to undergo transposition (111). Insertion by retrotransposition might then act to provide regulatory networks, or genetically/ epigenetically modify the developing embryo (88,89) during syngamy.

Repetitive elements enriched during early embryogenesis were also identified in this study. Upon fertilization repeat classes expressed from spermatogenesis switch to MER73 of the oocyte, which then later change during EGA to include an endogenous retrovirus (Figure 6). This suggests that the majority of zygotic repetitive element-containing RNAs are of maternal origin. During EGA, repeat expression again switches, to SVA-D and MLT2A1. Both ERVL retrotransposon LTRs have been implicated in mammalian embryonic development. HERVK is expected to increase in the morula and blastocyst stage human preimplantation embryos (112). However, we did not observe an enrichment for HERVK or HERVK LTRs in this set of expressed REs, a discrepancy that may be due to differing methods in library preparation and read assignment.

This study introduces a RE discovery algorithm (REDa) that identifies tissue and cell type specific expression in both exonic and intergenic REs. Expression patterns of REs were identified across human spermatogenesis, extending the current knowledge of the transcriptome in developing human sperm. In addition to observing considerable effects of poly(A^+^) enrichment, the sheer abundance of intergenic RNAs suggests that they play a large role in spermiogenesis. Of note, extensive expression of repetitive elements during spermatogenesis, suggests that perhaps these are driving spermatogenesis, while sperm-delivered repeat-derived RNAs may play more of a regulatory role in the human embryo.

## Supplementary Material

Supplementary Data

## References

[1] Lonsdale J., Thomas J., Salvatore M., Phillips R., Lo E., Shad S., Hasz R., Walters G., Garcia F., Young N. et al. The genotype-tissue expression (GTEx) project. Nat. Genet. 2013; 45:580. 23715323 10.1038/ng.2653 PMC4010069

[2] Uhlen M., Zhang C., Lee S., Sjöstedt E., Fagerberg L., Bidkhori G., Benfeitas R., Arif M., Liu Z., Edfors F. et al. A pathology atlas of the human cancer transcriptome. Science. 2017; 357. 10.1126/science.aan2507 28818916

[3] The GTEx Consortium 2018; Release V7 (dbGaP Accession phs000424.v7.p2) https://gtexportal.org/.

[4] EMBL-EBI 2018; Expression Atlas release 29 https://www.ebi.ac.uk/gxa/home.

[5] HPA 2018; Version 18.1 https://www.proteinatlas.org/.

[6] ENCODE 2018; Version 78.0 https://www.encodeproject.org/.

[7] Trapnell C., Williams B.A., Pertea G., Mortazavi A., Kwan G., van Baren M.J., Salzberg S.L., Wold B.J., Pachter L. Transcript assembly and quantification by RNA-Seq reveals unannotated transcripts and isoform switching during cell differentiation. Nat. Biotechnol. 2010; 28:511. 20436464 10.1038/nbt.1621 PMC3146043

[8] O’Leary N.A., Wright M.W., Brister J.R., Ciufo S., Haddad D., McVeigh R., Rajput B., Robbertse B., Smith-White B., Ako-Adjei D. et al. Reference sequence (RefSeq) database at NCBI: current status, taxonomic expansion, and functional annotation. Nucleic Acids Res. 2016; 44:D733–D745. 26553804 10.1093/nar/gkv1189 PMC4702849

[9] Harrow J., Frankish A., Gonzalez J.M., Tapanari E., Diekhans M., Kokocinski F., Aken B.L., Barrell D., Zadissa A., Searle S. et al. GENCODE: The reference human genome annotation for The ENCODE Project. Genome Res. 2012; 22:1760–1774. 22955987 10.1101/gr.135350.111 PMC3431492

[10] GENCODE 2018; GRCh38.p10, Release 26 https://www.gencodegenes.org/.

[11] Esteller M. Non-coding RNAs in human disease. Nat. Rev. Genet. 2011; 12:861. 22094949 10.1038/nrg3074

[12] Harries L.W. Long non-coding RNAs and human disease. Biochem. Soc. Trans. 2012; 40:902. 22817756 10.1042/BST20120020

[13] Salzman J., Chen R.E., Olsen M.N., Wang P.L., Brown P.O. Cell-Type specific features of circular RNA expression. PLos Genet. 2013; 9:e1003777. 24039610 10.1371/journal.pgen.1003777 PMC3764148

[14] Kim T.-K., Hemberg M., Gray J.M. Enhancer RNAs: aclass of long noncoding RNAs synthesized at enhancers. Cold Spring Harbor Perspect.Biol. 2015; 7:a018622. 10.1101/cshperspect.a018622 PMC4292161 25561718

[15] Djebali S., Davis C.A., Merkel A., Dobin A., Lassmann T., Mortazavi A., Tanzer A., Lagarde J., Lin W., Schlesinger F. et al. Landscape of transcription in human cells. Nature. 2012; 489:101. 22955620 10.1038/nature11233 PMC3684276

[16] Wu H., Nord A.S., Akiyama J.A., Shoukry M., Afzal V., Rubin E.M., Pennacchio L.A., Visel A. Tissue-Specific RNA expression marks Distant-Acting developmental enhancers. PLos Genet. 2014; 10:e1004610. 25188404 10.1371/journal.pgen.1004610 PMC4154669

[17] Kang H.J., Kawasawa Y.I., Cheng F., Zhu Y., Xu X., Li M., Sousa A.M.M., Pletikos M., Meyer K.A., Sedmak G. et al. Spatio-temporal transcriptome of the human brain. Nature. 2011; 478:483. 22031440 10.1038/nature10523 PMC3566780

[18] Svoboda P., Franke V., Schultz R.M. Lipshitz HD Current Topics in Developmental Biology. 2015; 113:Academic Press 305–349. 10.1016/bs.ctdb.2015.06.004 26358877

[19] Fagerberg L., Hallström B.M., Oksvold P., Kampf C., Djureinovic D., Odeberg J., Habuka M., Tahmasebpoor S., Danielsson A., Edlund K. et al. Analysis of the human tissue-specific expression by genome-wide integration of transcriptomics and antibody-based proteomics. Mol. Cell. Proteomics. 2014; 13:397–406. 24309898 10.1074/mcp.M113.035600 PMC3916642

[20] Sendler E., Johnson G.D., Mao S., Goodrich R.J., Diamond M.P., Hauser R., Krawetz S.A. Stability, delivery and functions of human sperm RNAs at fertilization. Nucleic Acids Res. 2013; 41:4104–4117. 23471003 10.1093/nar/gkt132 PMC3627604

[21] Johnson G.D., Sendler E., Lalancette C., Hauser R., Diamond M.P., Krawetz S.A. Cleavage of rRNA ensures translational cessation in sperm at fertilization. MHR: Basic Sci. Reprod. Med. 2011; 17:721–726. 10.1093/molehr/gar054 PMC3212859 21831882

[22] Johnson G.D., Mackie P., Jodar M., Moskovtsev S., Krawetz S.A. Chromatin and extracellular vesicle associated sperm RNAs. Nucleic Acids Res. 2015; 43:6847–6859. 26071953 10.1093/nar/gkv591 PMC4538811

[23] Jodar M., Soler-Ventura A., Oliva R. Semen proteomics and male infertility. J. Proteomics. 2017; 162:125–134. 27576136 10.1016/j.jprot.2016.08.018

[24] Sharma U., Conine C.C., Shea J.M., Boskovic A., Derr A.G., Bing X.Y., Belleannee C., Kucukural A., Serra R.W., Sun F. et al. Biogenesis and function of tRNA fragments during sperm maturation and fertilization in mammals. Science. 2016; 351:391. 26721685 10.1126/science.aad6780 PMC4888079

[25] Cossetti C., Lugini L., Astrologo L., Saggio I., Fais S., Spadafora C. Soma-to-Germline transmission of RNA in mice xenografted with human tumour Cells: Possible transport by exosomes. PLoS One. 2014; 9:e101629. 24992257 10.1371/journal.pone.0101629 PMC4081593

[26] Devanapally S., Ravikumar S., Jose A.M. Double-stranded RNA made in C. elegans neurons can enter the germline and cause transgenerational gene silencing. Proc. Natl. Acad. Sci. U.S.A. 2015; 112:2133. 25646479 10.1073/pnas.1423333112 PMC4343102

[27] Gòdia M., Swanson G., Krawetz S.A. A history of why Fathers’ RNA matters. Biol. Reprod. 2018; ioy007. 10.1093/biolre/ioy007 29514212

[28] Jodar M., Sendler E., Krawetz S.A. The protein and transcript profiles of human semen. Cell Tissue Res. 2016; 363:85–96. 26224537 10.1007/s00441-015-2237-1

[29] Krawetz S.A., Kruger A., Lalancette C., Tagett R., Anton E., Draghici S., Diamond M.P. A survey of small RNAs in human sperm. Hum. Reprod. 2011; 26:3401–3412. 21989093 10.1093/humrep/der329 PMC3212879

[30] Jodar M., Sendler E., Moskovtsev S.I., Librach C.L., Goodrich R., Swanson S., Hauser R., Diamond M.P., Krawetz S.A. Absence of sperm RNA elements correlates with idiopathic male infertility. Sci. Transl. Med. 2015; 7:295re296. 10.1126/scitranslmed.aab1287 PMC4721635 26157032

[31] Burl R.B., Clough S., Sendler E., Estill M., Krawetz S.A. Sperm RNA elements as markers of health. Syst. Biol. Reprod. Med. 2018; 64:25–38. 29199464 10.1080/19396368.2017.1393583

[32] Platts A.E., Dix D.J., Chemes H.E., Thompson K.E., Goodrich R., Rockett J.C., Rawe V.Y., Quintana S., Diamond M.P., Strader L.F. et al. Success and failure in human spermatogenesis as revealed by teratozoospermic RNAs. Hum. Mol. Genet. 2007; 16:763–773. 17327269 10.1093/hmg/ddm012

[33] Pertea M., Pertea G.M., Antonescu C.M., Chang T.-C., Mendell J.T., Salzberg S.L. StringTie enables improved reconstruction of a transcriptome from RNA-seq reads. Nat. Biotechnol. 2015; 33:290. 25690850 10.1038/nbt.3122 PMC4643835

[34] Xue Z., Huang K., Cai C., Cai L., Jiang C-y., Feng Y., Liu Z., Zeng Q., Cheng L., Sun Y.E. et al. Genetic programs in human and mouse early embryos revealed by single-cell RNA sequencing. Nature. 2013; 500:593. 23892778 10.1038/nature12364 PMC4950944

[35] Dang Y., Yan L., Hu B., Fan X., Ren Y., Li R., Lian Y., Yan J., Li Q., Zhang Y. et al. Tracing the expression of circular RNAs in human pre-implantation embryos. Genome Biol. 2016; 17:130. 27315811 10.1186/s13059-016-0991-3 PMC4911693

[36] Bates D., Mächler M., Bolker B., Walker S. Fitting linear mixed-effects models using lme4. J. Stat. Softw. 2015; 67:48.

[37] Benjamini Y., Hochberg Y. Controlling the false discovery rate: a practical and powerful approach to multiple testing. J. R. Stat. Soc. B (Methodological). 1995; 57:289–300.

[38] Flegel C., Vogel F., Hofreuter A., Schreiner B.S.P., Osthold S., Veitinger S., Becker C., Brockmeyer N.H., Muschol M., Wennemuth G. et al. Characterization of the olfactory receptors expressed in human spermatozoa. Front. Mol. Biosci. 2016; 2:73. 26779489 10.3389/fmolb.2015.00073 PMC4703994

[39] Shaffer J.P. Multiple hypothesis testing. Annu. Rev. Psychol. 1995; 46:561–584.

[40] Kumar L., Futschik E.M. Mfuzz: a software package for soft clustering of microarray data. Bioinformation. 2007; 2:5–7. 18084642 10.6026/97320630002005 PMC2139991

[41] Futschik M.E., carlisle B. Noise-robust soft clustering of gene expression time-course data. J. Bioinform. Comput. Biol. 2005; 03:965–988. 10.1142/s0219720005001375 16078370

[42] Jan S.Z., Vormer T.L., Jongejan A., Röling M.D., Silber S.J., de Rooij D.G., Hamer G., Repping S., van Pelt A.M.M. Unraveling transcriptome dynamics in human spermatogenesis. Development. 2017; 144:3659. 28935708 10.1242/dev.152413 PMC5675447

[43] Kalmar A., Wichmann B., Galamb O., Spisák S., Tóth K., Leiszter K., Tulassay Z., Molnár B. Gene expression analysis of normal and colorectal cancer tissue samples from fresh frozen and matched formalin-fixed, paraffin-embedded (FFPE) specimens after manual and automated RNA isolation. Methods. 2013; 59:S16–S19. 23036325 10.1016/j.ymeth.2012.09.011

[44] Thurman R.E., Rynes E., Humbert R., Vierstra J., Maurano M.T., Haugen E., Sheffield N.C., Stergachis A.B., Wang H., Vernot B. et al. The accessible chromatin landscape of the human genome. Nature. 2012; 489:75. 22955617 10.1038/nature11232 PMC3721348

[45] The E.P.C., Dunham I., Kundaje A., Aldred S.F., Collins P.J., Davis C.A., Doyle F., Epstein C.B., Frietze S., Harrow J. et al. An integrated encyclopedia of DNA elements in the human genome. Nature. 2012; 489:57. 22955616 10.1038/nature11247 PMC3439153

[46] Sabo P.J., Hawrylycz M., Wallace J.C., Humbert R., Yu M., Shafer A., Kawamoto J., Hall R., Mack J., Dorschner M.O. et al. Discovery of functional noncoding elements by digital analysis of chromatin structure. PNAS. 2004; 101:16837–16842. 15550541 10.1073/pnas.0407387101 PMC534745

[47] Wang J., Zhuang J., Iyer S., Lin X., Whitfield T.W., Greven M.C., Pierce B.G., Dong X., Kundaje A., Cheng Y. et al. Sequence features and chromatin structure around the genomic regions bound by 119 human transcription factors. Genome Res. 2012; 22:1798–1812. 22955990 10.1101/gr.139105.112 PMC3431495

[48] Gerstein M.B., Kundaje A., Hariharan M., Landt S.G., Yan K.-K., Cheng C., Mu X.J., Khurana E., Rozowsky J., Alexander R. et al. Architecture of the human regulatory network derived from ENCODE data. Nature. 2012; 489:91. 22955619 10.1038/nature11245 PMC4154057

[49] Dixon J.R., Selvaraj S., Yue F., Kim A., Li Y., Shen Y., Hu M., Liu J.S., Ren B. Topological domains in mammalian genomes identified by analysis of chromatin interactions. Nature. 2012; 485:376. 22495300 10.1038/nature11082 PMC3356448

[50] Rosenkranz D., Zischler H. proTRAC - a software for probabilistic piRNA cluster detection, visualization and analysis. BMC Bioinformatics. 2012; 13:5. 22233380 10.1186/1471-2105-13-5 PMC3293768

[51] Rosenkranz D. piRNA cluster database: a web resource for piRNA producing loci. Nucleic Acids Res. 2016; 44:D223–D230. 26582915 10.1093/nar/gkv1265 PMC4702893

[52] Smit A., Hubley R., Green P. RepeatMasker Open-3.0. 1996-2010; http://www.repeatmasker.org.

[53] Smit A.F. Identification of a new, abundant superfamily of mammalian LTR-transposons. Nucleic Acids Res. 1993; 21:1863–1872. 8388099 10.1093/nar/21.8.1863 PMC309426

[54] DFAM MSTC Long Terminal Repeat for ERVL-MaLR retrotransposon. 2018; http://dfam.org/entry/DF0001044.

[55] Franke V., Ganesh S., Karlic R., Malik R., Pasulka J., Horvat F., Kuzman M., Fulka H., Cernohorska M., Urbanova J. et al. Long terminal repeats power evolution of genes and gene expression programs in mammalian oocytes and zygotes. Genome Res. 2017; 27:1384–1394. 28522611 10.1101/gr.216150.116 PMC5538554

[56] Lysiak J.J. The role of tumor necrosis factor-alpha and interleukin-1 in the mammalian testis and their involvement in testicular torsion and autoimmune orchitis. Reprod. Biol. Endocrinol. 2004; 2:9. 15012831 10.1186/1477-7827-2-9 PMC404472

[57] Garcia T.X., Hofmann M.C. Regulation of germ line stem cell homeostasis. Anim. Reprod. 2015; 12:35–45. 28286576 PMC5341791

[58] Koch S., Acebron S.P., Herbst J., Hatiboglu G., Niehrs C. Post-transcriptional Wnt signaling governs epididymal sperm maturation. Cell. 2015; 163:1225–1236. 26590424 10.1016/j.cell.2015.10.029

[59] Kerr G.E., Young J.C., Horvay K., Abud H.E., Loveland K.L. Regulated Wnt/Beta-Catenin signaling sustains adult spermatogenesis in mice1. Biol. Reprod. 2014; 90:3. 24258210 10.1095/biolreprod.112.105809

[60] De Robertis E.M., Ploper D. Sperm motility requires Wnt/GSK3 stabilization of proteins. Dev. Cell. 2015; 35:401–402. 26609954 10.1016/j.devcel.2015.11.009

[61] Vidal F., Lopez P., Lopez-Fernandez L.A., Ranc F., Scimeca J.C., Cuzin F., Rassoulzadegan M. Gene trap analysis of germ cell signaling to Sertoli cells: NGF-TrkA mediated induction of Fra1 and Fos by post-meiotic germ cells. J. Cell Sci. 2001; 114:435–443. 11148144 10.1242/jcs.114.2.435

[62] Jin W., Tanaka A., Watanabe G., Matsuda H., Taya K. Effect of NGF on the motility and acrosome reaction of golden hamster spermatozoa *in vitro*. J. Reprod. Dev. 2010; 56:437–443. 20519834 10.1262/jrd.09-219n

[63] Michailov Y., Ickowicz D., Breitbart H. Zn2+-stimulation of sperm capacitation and of the acrosome reaction is mediated by EGFR activation. Dev. Biol. 2014; 396:246–255. 25446533 10.1016/j.ydbio.2014.10.009

[64] Shahar S., Hillman P., Lubart R., Ickowicz D., Breitbart H. Activation of sperm EGFR by light irradiation is mediated by reactive oxygen species. Photochem. Photobiol. 2014; 90:1077–1083. 24724551 10.1111/php.12281

[65] Ducummon C.C., Berger T. Localization of the Rho GTPases and some Rho effector proteins in the sperm of several mammalian species. Zygote. 2006; 14:249–257. 16822336 10.1017/S0967199406003790

[66] Irino Y., Ichinohe M., Nakamura Y., Nakahara M., Fukami K. Phospholipase Cδ4 associates with glutamate receptor interacting protein 1 in testis. J. Biochem. 2005; 138:451–456. 16272139 10.1093/jb/mvi135

[67] Ortiz-Ramírez C., Michard E., Simon A.A., Damineli D.S.C., Hernández-Coronado M., Becker J.D., Feijó J.A. Glutamate receptor-like channels are essential for chemotaxis and reproduction in mosses. Nature. 2017; 549:91. 28737761 10.1038/nature23478

[68] Margolin G., Khil P.P., Kim J., Bellani M.A., Camerini-Otero R.D. Integrated transcriptome analysis of mouse spermatogenesis. BMC Genomics. 2014; 15:39. 24438502 10.1186/1471-2164-15-39 PMC3906902

[69] Mulugeta Achame E., Baarends W.M., Gribnau J., Grootegoed J.A. Evaluating the relationship between spermatogenic silencing of the X chromosome and evolution of the Y chromosome in chimpanzee and human. PLoS One. 2010; 5:e15598. 21179482 10.1371/journal.pone.0015598 PMC3001880

[70] Sin H.-S., Ichijima Y., Koh E., Namiki M., Namekawa S.H. Human postmeiotic sex chromatin and its impact on sex chromosome evolution. Genome Res. 2012; 22:827–836. 22375025 10.1101/gr.135046.111 PMC3337429

[71] Ostermeier G.C., Miller D., Huntriss J.D., Diamond M.P., Krawetz S.A. Reproductive biology: delivering spermatozoan RNA to the oocyte. Nature. 2004; 429:154. 15141202 10.1038/429154a

[72] Krawetz S.A. Paternal contribution: new insights and future challenges. Nat. Rev. Genet. 2005; 6:633–642. 16136654 10.1038/nrg1654

[73] Sachani S. Nucleoporin-mediated regulation of the Kcnq1ot1 imprinted domain. 2016; University of Western Ontario, Electronic Thesis and Dissertation Repository.

[74] Tsukamoto S., Kuma A., Mizushima N. The role of autophagy during the oocyte-to-embryo transition. Autophagy. 2008; 4:1076–1078. 18849666 10.4161/auto.7065

[75] Werber M., Wittler L., Timmermann B., Grote P., Herrmann B.G. The tissue-specific transcriptomic landscape of the mid-gestational mouse embryo. Development. 2014; 141:2325–2330. 24803591 10.1242/dev.105858

[76] Kojima Y., Tam O.H., Tam P.P.L. Timing of developmental events in the early mouse embryo. Semin. Cell Dev. Biol. 2014; 34:65–75. 24954643 10.1016/j.semcdb.2014.06.010

[77] Cao S., Han J., Wu J., Li Q., Liu S., Zhang W., Pei Y., Ruan X., Liu Z., Wang X. et al. Specific gene-regulation networks during the pre-implantation development of the pig embryo as revealed by deep sequencing. BMC Genomics. 2014; 15:4. 24383959 10.1186/1471-2164-15-4 PMC3925986

[78] Fan X., Zhang X., Wu X., Guo H., Hu Y., Tang F., Huang Y. Single-cell RNA-seq transcriptome analysis of linear and circular RNAs in mouse preimplantation embryos. Genome Biol. 2015; 16:148. 26201400 10.1186/s13059-015-0706-1 PMC4511241

[79] Yan L., Yang M., Guo H., Yang L., Wu J., Li R., Liu P., Lian Y., Zheng X., Yan J. et al. Single-cell RNA-Seq profiling of human preimplantation embryos and embryonic stem cells. Nat. Struct. Mol. Biol. 2013; 20:1131. 23934149 10.1038/nsmb.2660

[80] Team, R.C. R: A language and environment for statistical computing. R Foundation for Statistical Computing. 2018; Vienna.

[81] Sozen B., Can A., Demir N. Cell fate regulation during preimplantation development: A view of adhesion-linked molecular interactions. Dev. Biol. 2014; 395:73–83. 25176042 10.1016/j.ydbio.2014.08.028

[82] Miller D. Confrontation, consolidation, and Recognition: The Oocyte's perspective on the incoming sperm. Cold Spring Harbor Perspect.Med. 2015; 5:a023408. 10.1101/cshperspect.a023408 PMC4526728 25957313

[83] Youngson N.A., Lecomte V., Maloney C.A., Leung P., Liu J., Hesson L.B., Luciani F., Krause L., Morris M.J. Obesity-induced sperm DNA methylation changes at satellite repeats are reprogrammed in rat offspring. Asian J. Androl. 2016; 18:930–936. 26608942 10.4103/1008-682X.163190 PMC5109891

[84] Shea J.M., Serra R.W., Carone B.R., Shulha H.P., Kucukural A., Ziller M.J., Vallaster M.P., Gu H., Tapper A.R., Gardner P.D. et al. Genetic and epigenetic variation, but not diet, shape the sperm methylome. Dev Cell. 2015; 35:750–758. 26702833 10.1016/j.devcel.2015.11.024 PMC4691283

[85] Catasti P., Chen X., Mariappan S.V.S., Bradbury E.M., Gupta G. DNA repeats in the human genome. Genetica. 1999; 106:15–36. 10710707 10.1023/a:1003716509180

[86] Yaron Y., Kramer J.A., Gyi K., Ebrahim S.A., Evans M.I., Johnson M.P., Krawetz S.A. Centromere sequences localize to the nuclear halo of human spermatozoa. Int. J. Androl. 1998; 21:13–18. 9639147 10.1046/j.1365-2605.1998.00085.x

[87] Linnemann A. Ph.D. Thesis: Analysis of Nuclear Scaffold/Matrix Attachment: The Role of Genome Organization in Transcription. 2009; Wayne State University, Center for Molecular Medicine and Genetics 184.

[88] Spadafora C. Sperm-mediated stransgenerational inheritance. Front.Microbiol. 2017; 8:2401. 29255455 10.3389/fmicb.2017.02401 PMC5722983

[89] Spadafora C. A LINE‐1–encoded reverse transcriptase–dependent regulatory mechanism is active in embryogenesis and tumorigenesis. Ann. N. Y. Acad. Sci. 2015; 1341:164–171. 25586649 10.1111/nyas.12637

[90] Giordano R., Magnano A.R., Zaccagnini G., Pittoggi C., Moscufo N., Lorenzini R., Spadafora C. Reverse transcriptase activity in mature spermatozoa of mouse. J. Cell Biol. 2000; 148:1107. 10725323 10.1083/jcb.148.6.1107 PMC2174319

[91] Theunissen T.W., Friedli M., He Y., Planet E., O’Neil R.C., Markoulaki S., Pontis J., Wang H., Iouranova A., Imbeault M. et al. Molecular criteria for defining the naive human pluripotent state. Cell Stem Cell. 2016; 19:502–515. 27424783 10.1016/j.stem.2016.06.011 PMC5065525

[92] Raz T., Kapranov P., Lipson D., Letovsky S., Milos P.M., Thompson J.F. Protocol dependence of Sequencing-Based gene expression measurements. PLoS One. 2011; 6:e19287. 21573114 10.1371/journal.pone.0019287 PMC3089619

[93] Schrom E.-M., Moschall R., Schuch A., Bodem J. Maramorosch K, Murphy FA Advances in Virus Research. 2013; 85:Academic Press 1–24. 10.1016/B978-0-12-408116-1.00001-X 23439022

[94] Borodulina O.R., Golubchikova J.S., Ustyantsev I.G., Kramerov D.A. Polyadenylation of RNA transcribed from mammalian SINEs by RNA polymerase III: complex requirements for nucleotide sequences. Biochim. Biophys. Acta (BBA) - Gene Regul. Mech. 2016; 1859:355–365. 10.1016/j.bbagrm.2015.12.003 26700565

[95] Krane D.E., Hardison R.C. Short interspersed repeats in rabbit DNA can provide functional polyadenylation signals. Mol. Biol. Evol. 1990; 7:1–8. 2299978 10.1093/oxfordjournals.molbev.a040583

[96] Heui-Soo K. Genomic impact, chromosomal distribution and transcriptional regulation of HERV elements. Mol. Cells. 2012; 33:539–544. 22562360 10.1007/s10059-012-0037-y PMC3887755

[97] Curinha A., Oliveira Braz S., Pereira-Castro I., Cruz A., Moreira A. Implications of polyadenylation in health and disease. Nucleus. 2014; 5:508–519. 25484187 10.4161/nucl.36360 PMC4615197

[98] Jachowicz J.W., Bing X., Pontabry J., Bošković A., Rando O.J., Torres-Padilla M.-E. LINE-1 activation after fertilization regulates global chromatin accessibility in the early mouse embryo. Nat. Genet. 2017; 49:1502. 28846101 10.1038/ng.3945

[99] Trapnell C., Roberts A., Goff L., Pertea G., Kim D., Kelley D.R., Pimentel H., Salzberg S.L., Rinn J.L., Pachter L. Differential gene and transcript expression analysis of RNA-seq experiments with TopHat and Cufflinks. Nat. Protoc. 2012; 7:562. 22383036 10.1038/nprot.2012.016 PMC3334321

[100] Jodar M., Sendler E., Moskovtsev S.I., Librach C.L., Goodrich R., Swanson S., Hauser R., Diamond M.P., Krawetz S.A. Response to Comment on “Absence of sperm RNA elements correlates with idiopathic male infertility”. Sci. Transl. Med. 2016; 8:353tr351. 10.1126/scitranslmed.aaf4550 27559098

[101] Ntostis P., Carter D., Iles D., Huntriss J., Tzetis M., Miller D. Potential sperm contributions to the murine zygote predicted by in silico analysis. Reproduction. 2017; 154:777–788. 28916718 10.1530/REP-17-0097

[102] Lee M.T., Bonneau A.R., Giraldez A.J. Zygotic genome activation during the maternal-to-zygotic transition. Annu. Rev. Cell Dev. Biol. 2014; 30:581–613. 25150012 10.1146/annurev-cellbio-100913-013027 PMC4303375

[103] Jodar M., Selvaraju S., Sendler E., Diamond M.P., Krawetz S.A. for the Reproductive Medicine, N. The presence, role and clinical use of spermatozoal RNAs. Hum. Reprod. Update. 2013; 19:604–624. 23856356 10.1093/humupd/dmt031 PMC3796946

[104] Wang G., Guo Y., Zhou T., Shi X., Yu J., Yang Y., Wu Y., Wang J., Liu M., Chen X. et al. In-depth proteomic analysis of the human sperm reveals complex protein compositions. J. Proteomics. 2013; 79:114–122. 23268119 10.1016/j.jprot.2012.12.008

[105] Davidson E.H., Britten R.J. Regulation of gene Expression: Possible role of repetitive sequences. Science. 1979; 204:1052–1059. 451548 10.1126/science.451548

[106] Britten R.J., Davidson E.H. Gene regulation for higher cells: a theory. Science. 1969; 165:349. 5789433 10.1126/science.165.3891.349

[107] Johnson G.D., Jodar M., Pique-Regi R., Krawetz S.A. Nuclease footprints in sperm project past and future chromatin regulatory events. Sci. Rep. 2016; 6:25864. 27184706 10.1038/srep25864 PMC4869110

[108] Ioannou D., Millan N.M., Jordan E., Tempest H.G. A new model of sperm nuclear architecture following assessment of the organization of centromeres and telomeres in three-dimensions. Sci. Rep. 2017; 7:41585. 28139771 10.1038/srep41585 PMC5282497

[109] Velazquez Camacho O., Galan C., Swist-Rosowska K., Ching R., Gamalinda M., Karabiber F., De La Rosa-Velazquez I., Engist B., Koschorz B., Shukeir N. et al. Major satellite repeat RNA stabilize heterochromatin retention of Suv39h enzymes by RNA-nucleosome association and RNA:DNA hybrid formation. eLife. 2017; 6:e25293. 28760199 10.7554/eLife.25293 PMC5538826

[110] Scherthan H., Schöfisch K., Dell T., Illner D. Contrasting behavior of heterochromatic and euchromatic chromosome portions and pericentric genome separation in pre-bouquet spermatocytes of hybrid mice. Chromosoma. 2014; 123:609–624. 25119530 10.1007/s00412-014-0479-4 PMC4226931

[111] Spadafora C. The “evolutionary field” hypothesis. Non-Mendelian transgenerational inheritance mediates diversification and evolution. Prog. Biophys. Mol. Biol. 2018; 134:27–37. 29223657 10.1016/j.pbiomolbio.2017.12.001

[112] Grow E.J., Flynn R.A., Chavez S.L., Bayless N.L., Wossidlo M., Wesche D.J., Martin L., Ware C.B., Blish C.A., Chang H.Y. et al. Intrinsic retroviral reactivation in human preimplantation embryos and pluripotent cells. Nature. 2015; 522:221. 25896322 10.1038/nature14308 PMC4503379

