# Supplementary material for: RNA element discovery from germ cell to blastocyst
Source: Nucleic Acids Res. 2018 Dec 21;47(5):2263–75. doi: 10.1093/nar/gky1223 (PMC6411832; doi:10.1093/nar/gky1223)
Supplement: Supplementary Data [file gky1223_supplemental_files.zip › Supplemental_appendix_A.docx]

**Supplemental Files:**

**Appendix:**

**Computational Methods:**

**Mfuzz clustering**

Mfuzz is an R package designed for soft clustering of gene expression time-series data (1). The samples used in clustering were the Jan et al. spermatogenesis libraries (2), as well as a set of 7 ejaculated sperm samples from fertile males (3). The median expression value for the 7 mature sperm samples was used to represent the mature sperm samples as a single value. The RE dataset was then composed of a single library for A_dark_ SSCs, A_pale_ SSCs, Leptotyne/Zygotene, Early Pachytene, Late Pachytene, and Round spermatids, while the library for ejaculated sperm is the median expression value for 7 fertile males. In a step intended to remove universally lowly expressed REs, REs were processed to remove those which did not exceed 25 RPKM in at least one sample. Mfuzz clustering was performed, generating 20 cluster patterns, with a minimal membership of 0.7 required for inclusion in a pattern.

**Processing bam files**

The RE discovery algorithm is designed to be run entirely in R, with the user providing aligned reads in BAM file format. To conserve memory, the BAM files are first converted to bigWig format. The user is expected to use the helper function “generatebw” to generate both the required bigWigs as well as the text file containing the number of aligned reads per sample. However, the required bigWigs can also be generated by converting BAM files to bedgraph format, using the bedtools tool genomeCoverageBed, with the parameters “-split –bg”, and subsequently bigwig format, using the bedGraphToBigWig program (available from the UCSC Genome Browser utilities).

**Collapsing redundant exons**

Gene isoforms and transcripts often share overlapping exons and UTRs. In the RE discovery tool, overlapping annotated regions (here described as exons, regardless of the coding potential) on the same strand are collapsed into a single loci, designated as “exonic”. This singular representation of multi-exonic transcripts is not conducive to isoform discovery, but works well for fragmented RNAs, as is seen in spermatozoa or formalin-fixed paraffin embedded (FFPE) tissues. The “prepareExons” function collapses exons using the “bedR” package.

**Discovering expressed regions**

The initial step in discovering expressed regions of the genome is performed in the “findRE” function. The user needs to provide their genome of interest in BSgenome format, and a gene annotation file in GTF format. Although the current study performed RE discovery on the hg38 build of the human genome, the algorithm is adaptable for different species and genome builds. There is no restriction on the format of chromosome names used in the genome. However, later annotation steps are coded to be used with ensembl gene annotations available through the R package ‘biomaRt’, and thus it is recommended that the GTF file uses ensembl gene IDs (please see <https://www.gencodegenes.org/releases/current.html> for examples). The genome is first processed by binning into 10 bp regions. Coverage across each 10 bp bin is calculated and bins overlapping an annotated region in the GTF file (here described as exons, regardless of the coding potential) are removed from consideration. The remaining genomic bins are compared to the designated library size-normalized threshold mu (µ), with those bins equal to or exceeding the coverage required in µ retained as a novel RNA element (RE). This threshold µ, for a theoretical RNA-seq library with 3 million reads, would require a mean coverage of 7.5 reads for a given 10 bp bin in order to label the bin as expressed. REs within 100 bp of one another are merged into a single new RE. It is important to note that the default parameters used by the “findRE” function retain expressed regions if they are present in at least one sample. REs defined across each sample are subsequently merged with the “combineRE” function, in which the REs from each iteration are concatenated into a single GRanges object. The concatenated object is then reduced, merging overlapping and adjacent (within 50 bp) REs.

**Annotating REs**

Once the newly discovered REs are generated, they need to be annotated according to their genomic position, which is achieved in the “annotateRE” function. Intronic regions are defined according to the user-supplied GTF file, followed by identifying novel REs that overlap an intron. The distance between any non-intronic novel REs and exons is then calculated and used to define non-intronic novel REs as Near-Exon (less than 10kb from an exon) or Orphan (greater than or equal to 10 kb from an exon).

In the human genome, poorly annotated genes have occasionally been observed to have transcription beyond their designated borders. In an optional step, performed with the “extendExon” function, Near-Exon REs within a default 20 bp of an exon are examined. The read coverage across Near-Exon REs, in 10 bp increments, are compared to the average read coverage of the adjacent exon. If the read coverage in the Near-Exon RE is increased or decreased by less than 50% of the average read coverage of the adjacent exon, the novel RE is merged with the adjacent exon. This process is repeated until no more 10 bp bins of the Near-Exon RE remain, or the Near-Exon RE coverage changes by more than 50% of the average read coverage of the adjacent exon.

In order to provide the user with the common gene symbol of each ensembl gene ID, the function “annotateFinal” is provided. The “biomaRt” package is used to transform the ensembl gene ID into the common gene symbol. The output is a complete bed file of exonic and novel REs, along with the common gene symbol(s) and ensembl gene id(s) of the REs. This bed file can subsequently be used in expression analysis.

**Supplemental Figures**

**
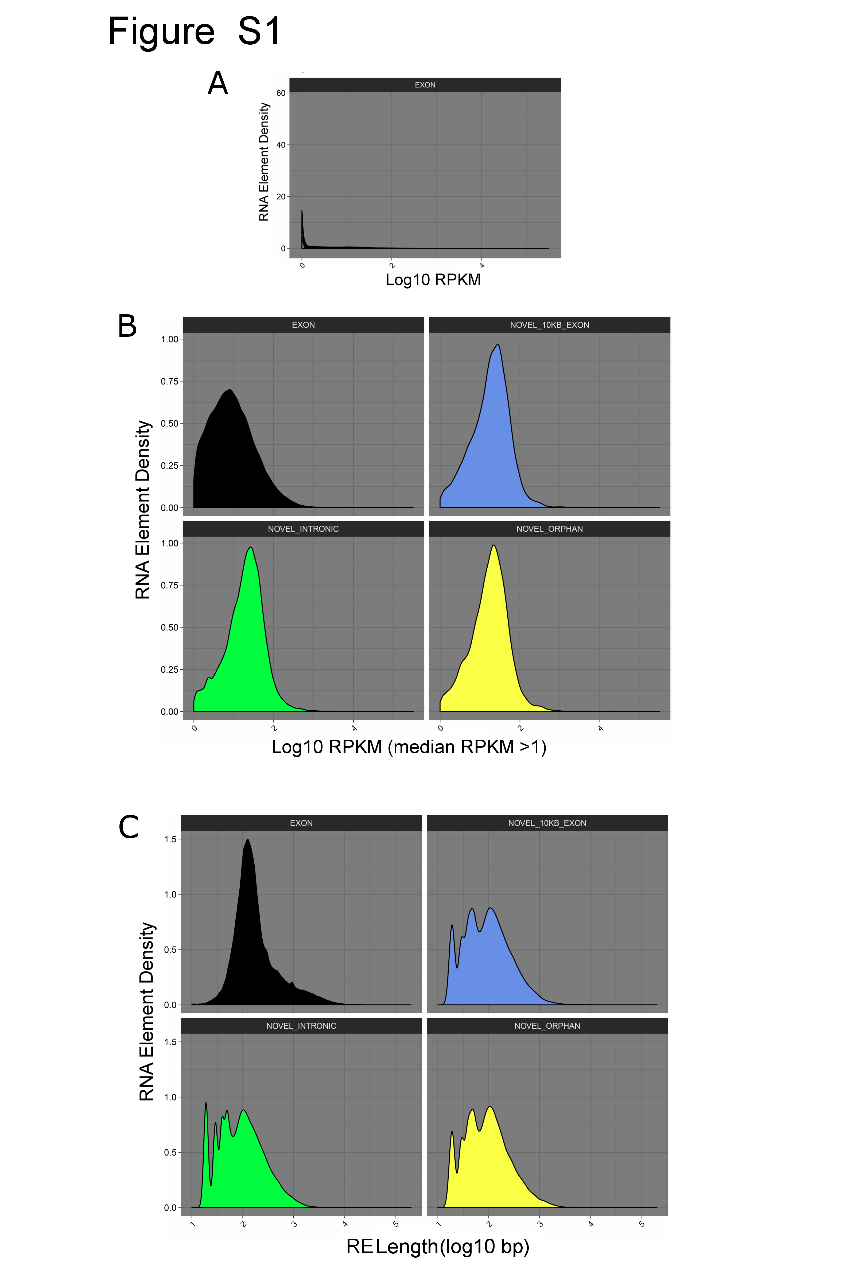
**

**Figure S1. Expression and length distribution of REs.** (A) Expression distribution of Exonic REs for with all sample types, with all sample types overlaid. The X-axis represents the log10 transformation of the RE’s expression in RPKM +1 RPKM. Across any single sample type, the majority of REs are lowly expressed. (B). Expression distribution of all RE classes for sperm total RNA samples, after elimination of lowly expressed (median expression <1 RPKM) REs. The X-axis represents the log10 transformation of the RE’s expression in RPKM +1 RPKM (C) Length distribution of all REs, according to the RE classes. X-axis represents the log10 transformation of the RE’s width in base pairs.

**
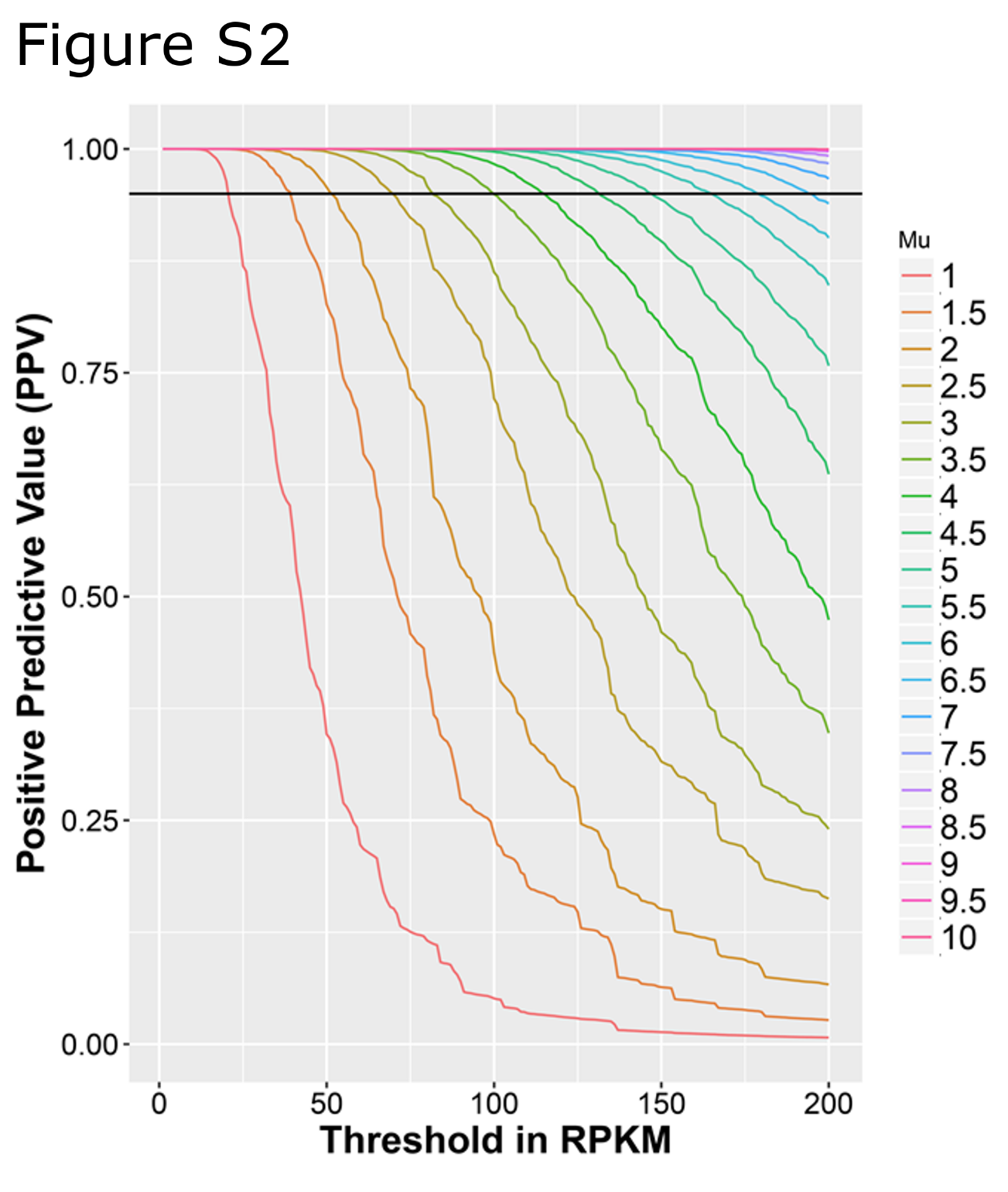
**

**Figure S2. Background noise for read coverage thresholds.** X-axis represents the experimental threshold for calling a RE as “true positive (TP)”. The corresponding Positive Predictive Value (PPV) is calculated as (TP/(TP+FP)). The PPV curve is provided for levels of μ from 1 RPM to 10 RPM. The X-axis represents the expression threshold required for assigning a RE as expressed, and ranges from 1 to 200 RPKM. A PPV of 0.95 (corresponding to a False Discovery Rate (FDR) of 5%), is shown as a black line.

**
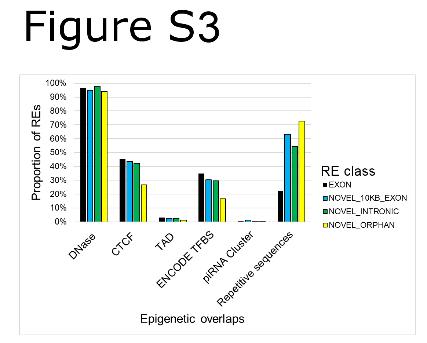
Figure S3. Overlap of REs with epigenetic marks and regulatory genomic sequences.** The proportion of each RE class classified as overlapping a given epigenetic mark or genomic sequence is indicated on the Y-axis, with the type of epigenetic mark or genomic sequence indicated on the X-axis. The type of RE class is indicated with Exonic REs in black, Near-exon REs in light blue, Intronic REs in green, and Orphan REs in yellow. “DNase” indicates overlaps within DNase I Hypersensitivity Peak Clusters from ENCODE (95 cell types) or 5 kb of a cluster. “CTCF” indicates overlaps within CTCF binding sites in the GM12878 cell line (determined from ChIP-seq) or 5 kb of a binding site. “TAD” indicates overlaps within 5 kb of a topologically associating domain (TAD) ending site. “ENCODE TFBS” indicates overlaps with Transcription Factor Binding Sites (TFBS) from Encode (ENCODE Mar 2012 Freeze). “piRNA Cluster” indicates overlaps with piRNAs in the human genome. “Repetitive sequences” indicates overlaps with UCSC’s Repeatmasker track (last updated 2014-01-10).

**
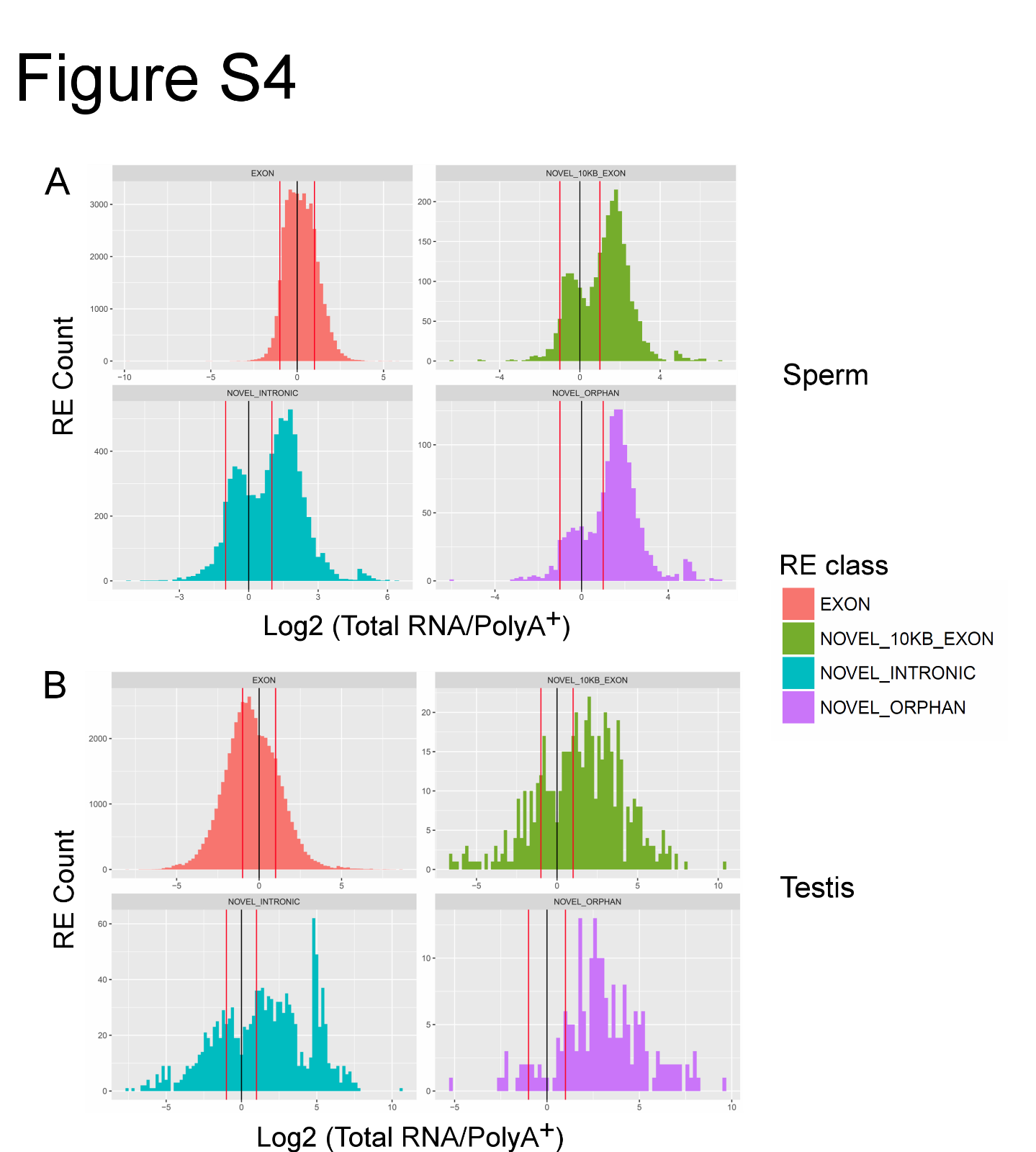
Figure S4. Total RNA libraries are enriched for Novel REs in sperm and testes.** (A) Histogram of fold changes (log2 transformed) are shown for (A) mature sperm and (B) testes samples. The X-axis indicates the Log2 of the Total RNA/polyA^+^ ratio, with positive change and negative change representing enrichment in total RNA and poly(A^+^) libraries, respectively. Note the novel REs all exhibit a shift to the right, indicating increased expression in total RNA samples.

**
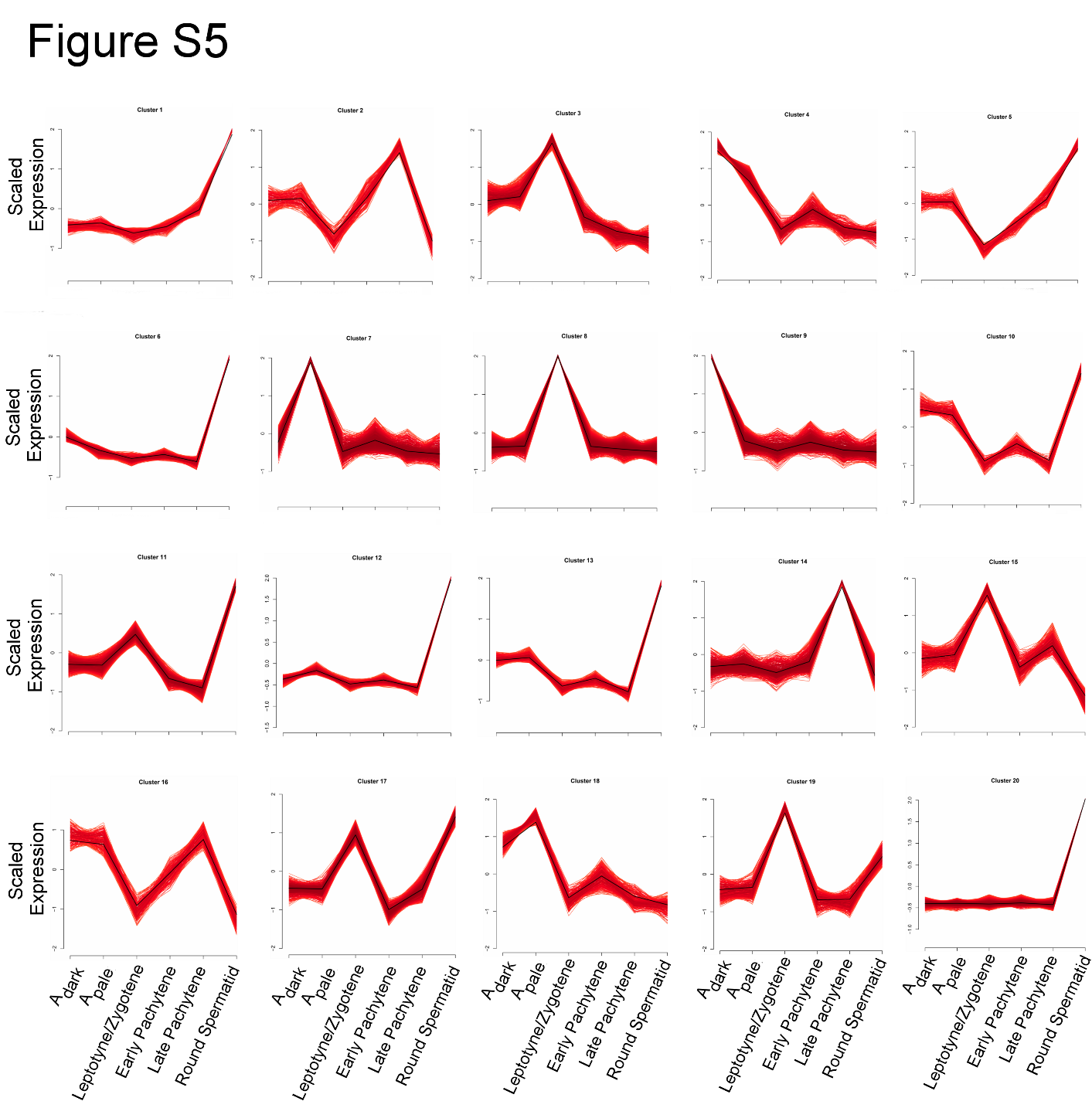
Figure S5. Mfuzz clusters for 6 spermatogenic stages**. Scaled expression changes across the pre-meiotic, meiotic, and post-meiotic cell types provided in Jan et al. Red lines indicate the expression changes of individual REs, while a black line indicates the underlying expression pattern. Note that the RE patterns broadly recapitulate those published by Jan et al.


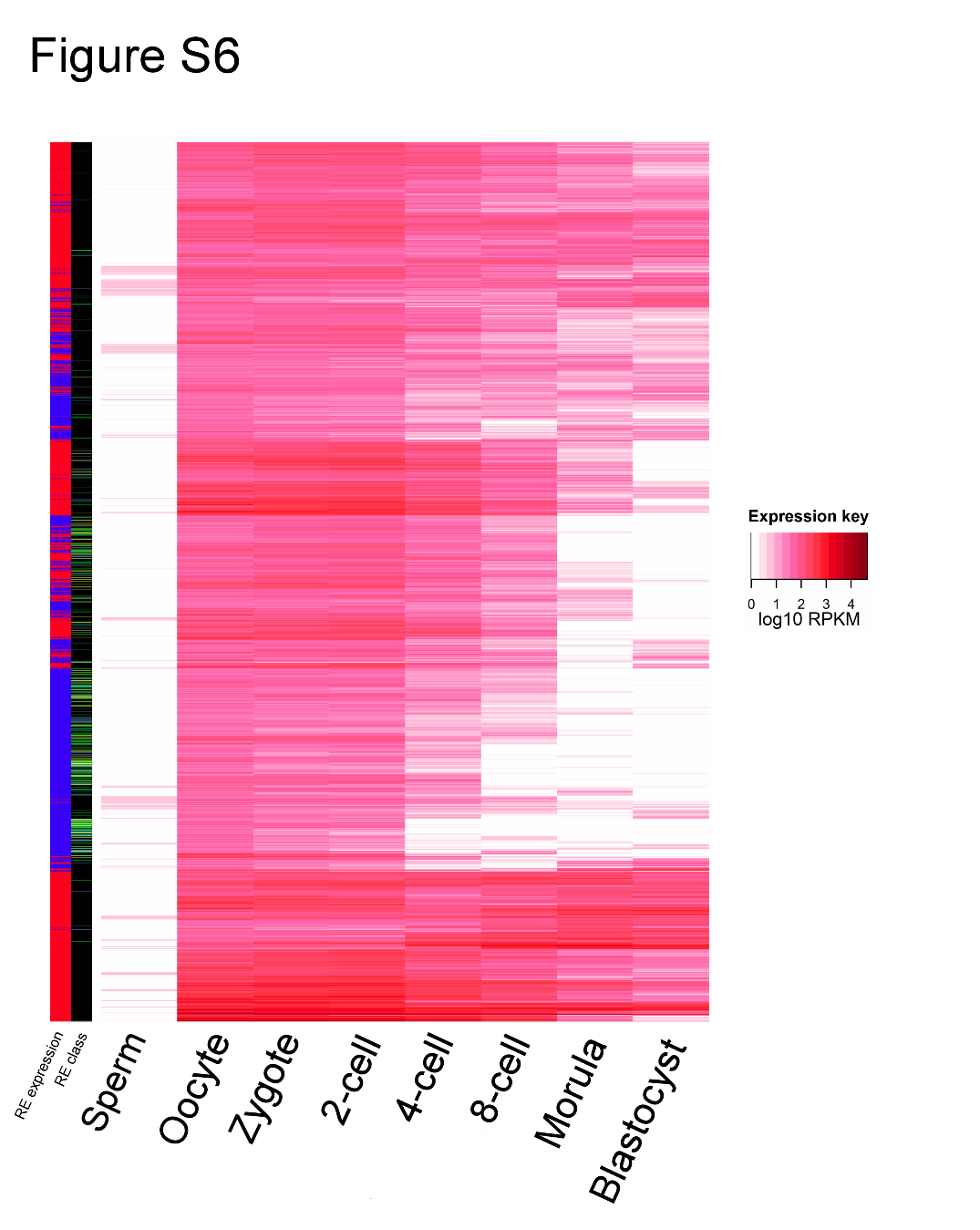


**Figure S6. Expression heatmap of maternally derived REs**. The overall expression level is represented in “RE expression”, with red indicating a median expression exceeding 25 RPKM. RE class, shown adjacent to the RE expression column, shows Exonic REs in black, Intronic REs in green, Near-exon REs in light blue, and Orphan REs in yellow. The REs presented are supplied by the oocyte to the zygote (Sperm < 2 RPKM; Oocyte > 25 RPKM; Zygote > 10 RPKM)

**
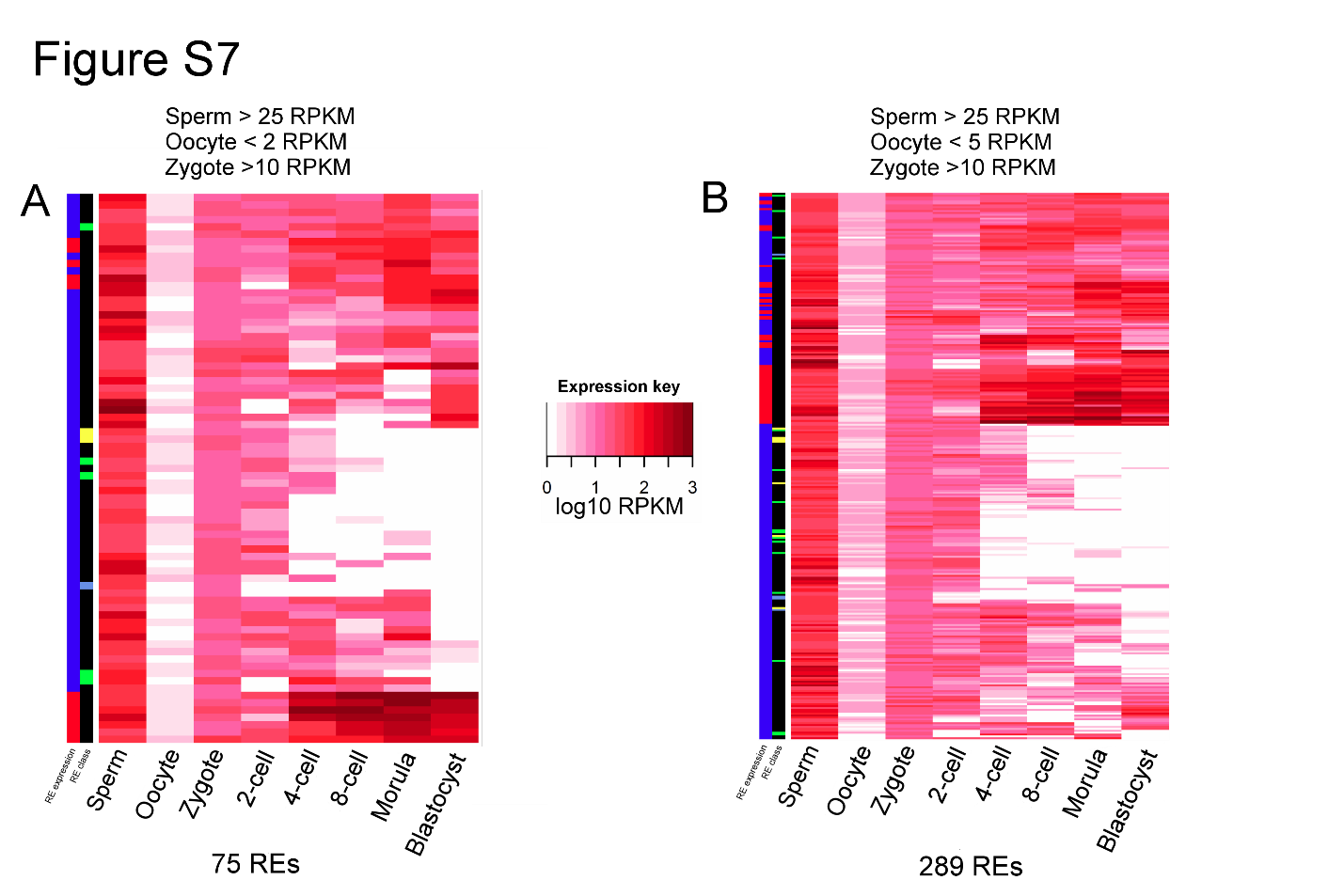
**

**Figure S7**. **Expression heatmap of paternally derived REs.** The overall expression level is represented in “RE expression”, with red indicating a median expression exceeding 25 RPKM. RE class, shown adjacent to the RE expression column, shows Exonic REs in black, Intronic REs in green, Near-exon REs in light blue, and Orphan REs in yellow. The REs presented are supplied by the sperm to the zygote, with strong sperm preference (A) Sperm > 25 RPKM; Oocyte < 2 RPKM; Zygote > 10 RPKM and moderate sperm preference (B) Sperm > 25 RPKM; Oocyte < 5 RPKM ; Zygote > 10 RPKM

**
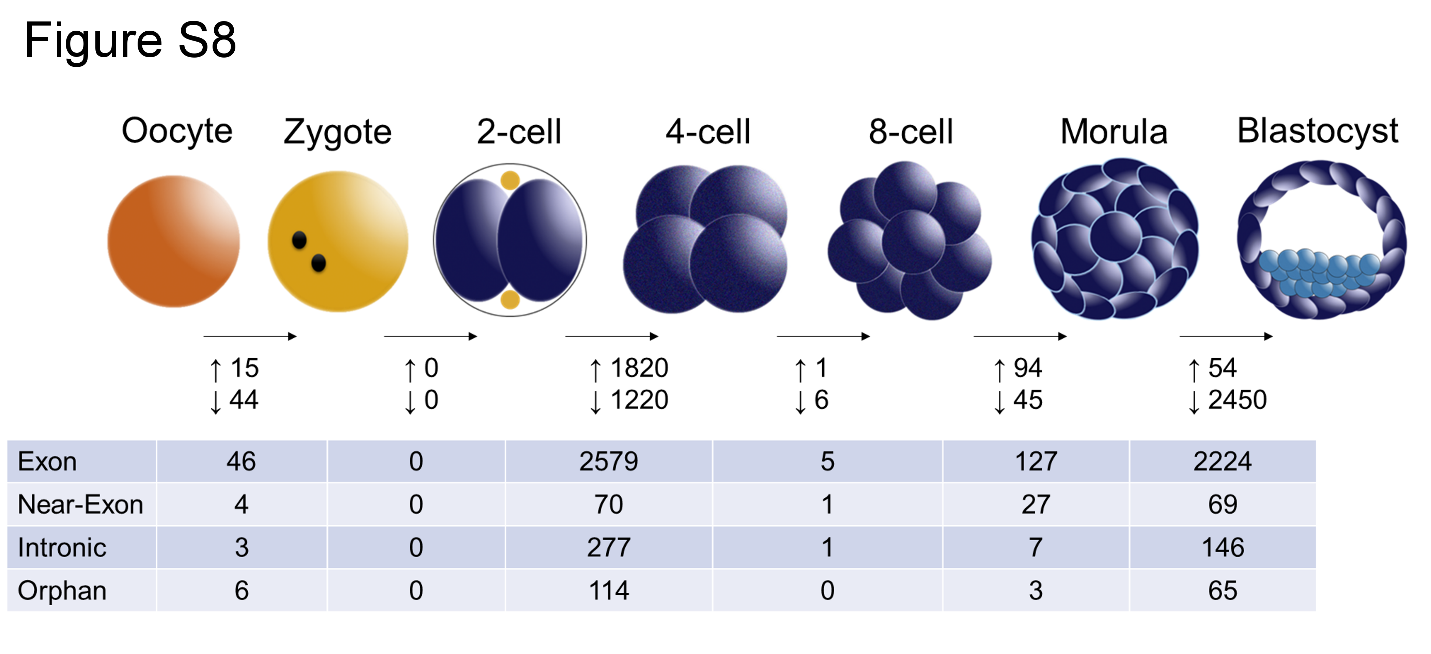
**

**Figure S8. Differential RE expression across early embryonic development**. The count of differential up- and down-regulated REs as embryogenesis proceeds is shown below the diagrams of cell types. The annotation classes of the total differential REs are summarized in the bottom table.


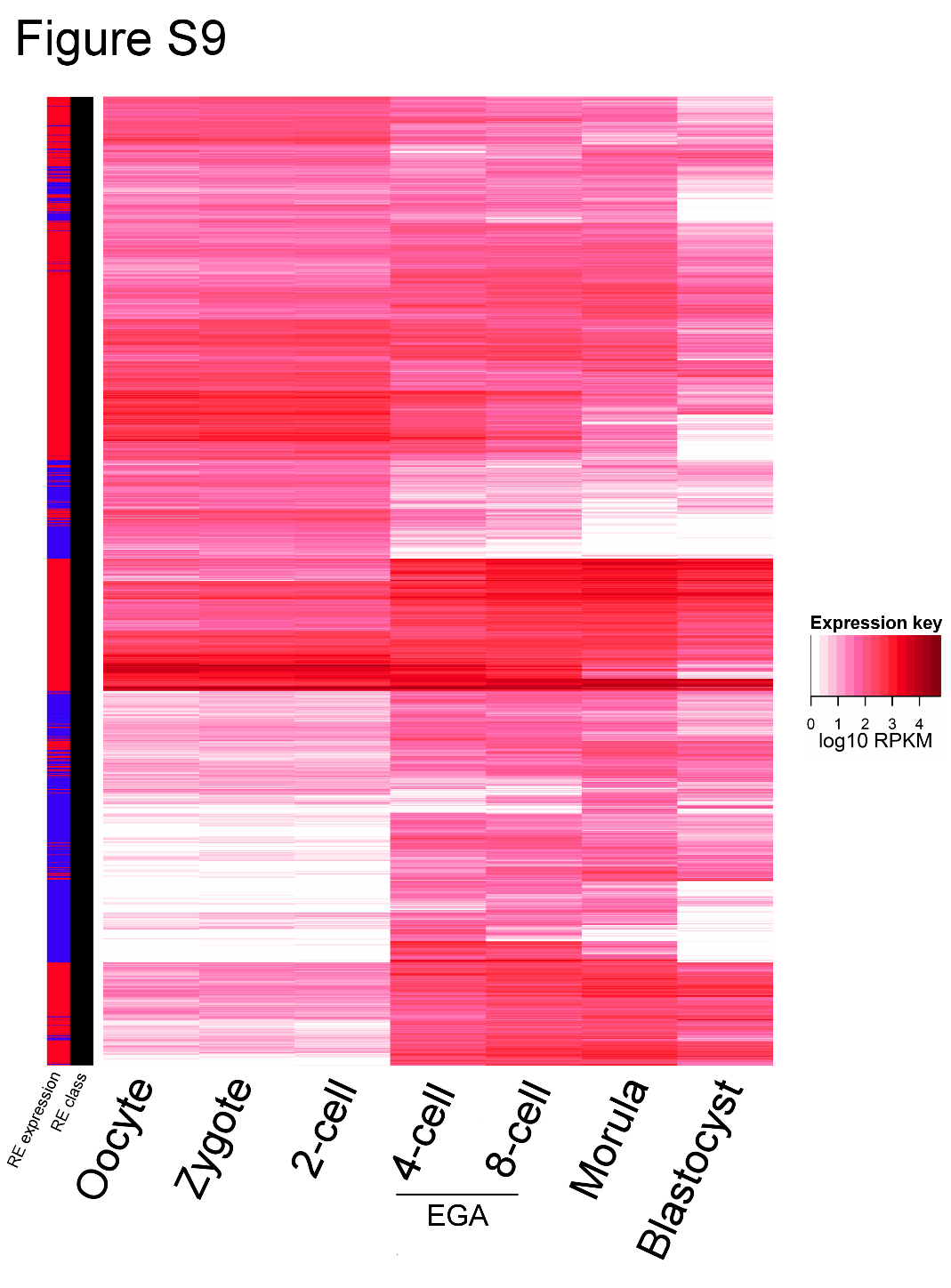


**Figure S9. Expression heatmap of differentially expressed exonic REs across early embryogenesis**. The overall expression level is represented in “RE expression”, with red indicating a median expression exceeding 25 RPKM. RE class, shown adjacent to the RE expression column, shows Exonic REs in black. The REs presented are differentially expressed across at least one stage.

**Supplemental Table Legends**

**All Tables are appended as xlsx files**

**Table S1.** Publication information on datasets used in the study.

**Table S2.** Complete RE loci and gene associations

**Table S3.** Benchmark of RE loci with Stringtie and Cufflinks

**Table S4.** Repeat distribution of polyA-enriched orphan REs

**Table S5.** Gene ontology of polyA selection in oocytes and embryo

**Table S6.** Expression values for Jan et. Al. and Serono sperm samples

**Table S7.** RE class distribution of Mfuzz clusters

**Table S8.** Genomatix Gene Ontology for REs expressed in Round Spermatids and Mature Spermatozoa

**Table S9.** Location and expression of paternally transmitted REs

**References**

1. Kumar, L. and E. Futschik, M. (2007) Mfuzz: A software package for soft clustering of microarray data. *Bioinformation*, **2**, 5-7.

2. Jan, S.Z., Vormer, T.L., Jongejan, A., Röling, M.D., Silber, S.J., de Rooij, D.G., Hamer, G., Repping, S. and van Pelt, A.M.M. (2017) Unraveling transcriptome dynamics in human spermatogenesis. *Development*, **144**, 3659.

3. Jodar, M., Sendler, E., Moskovtsev, S.I., Librach, C.L., Goodrich, R., Swanson, S., Hauser, R., Diamond, M.P. and Krawetz, S.A. (2015) Absence of sperm RNA elements correlates with idiopathic male infertility. *Science Translational Medicine*, **7**, 295re296.
