# Supplementary figures and images for: RNA element discovery from germ cell to blastocyst
Source: Nucleic Acids Res. 2018 Dec 21;47(5):2263–75. doi: 10.1093/nar/gky1223 (PMC6411832; doi:10.1093/nar/gky1223)

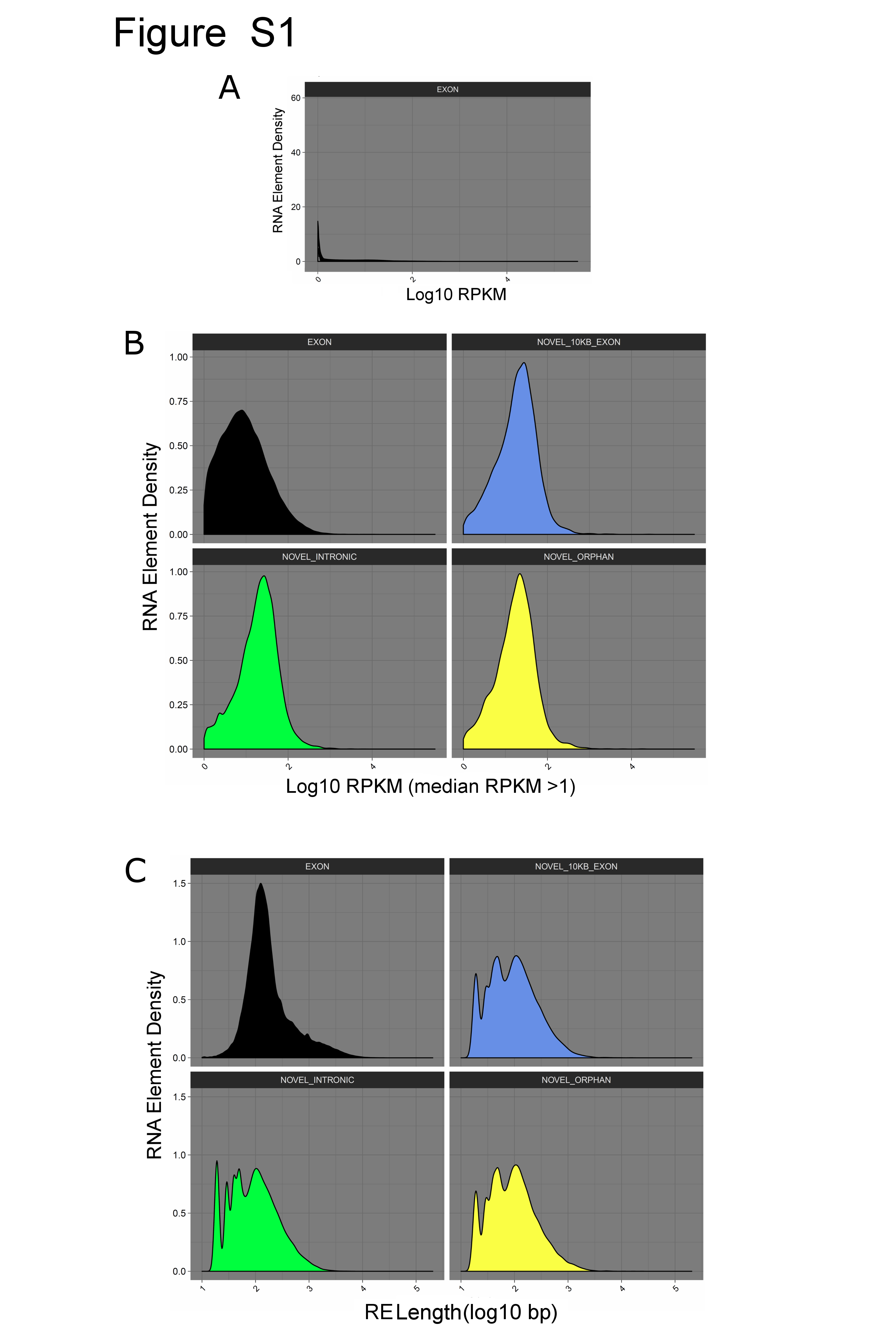

Supplement: Supplementary Data [file gky1223_supplemental_files.zip › Figure_S1.tiff]

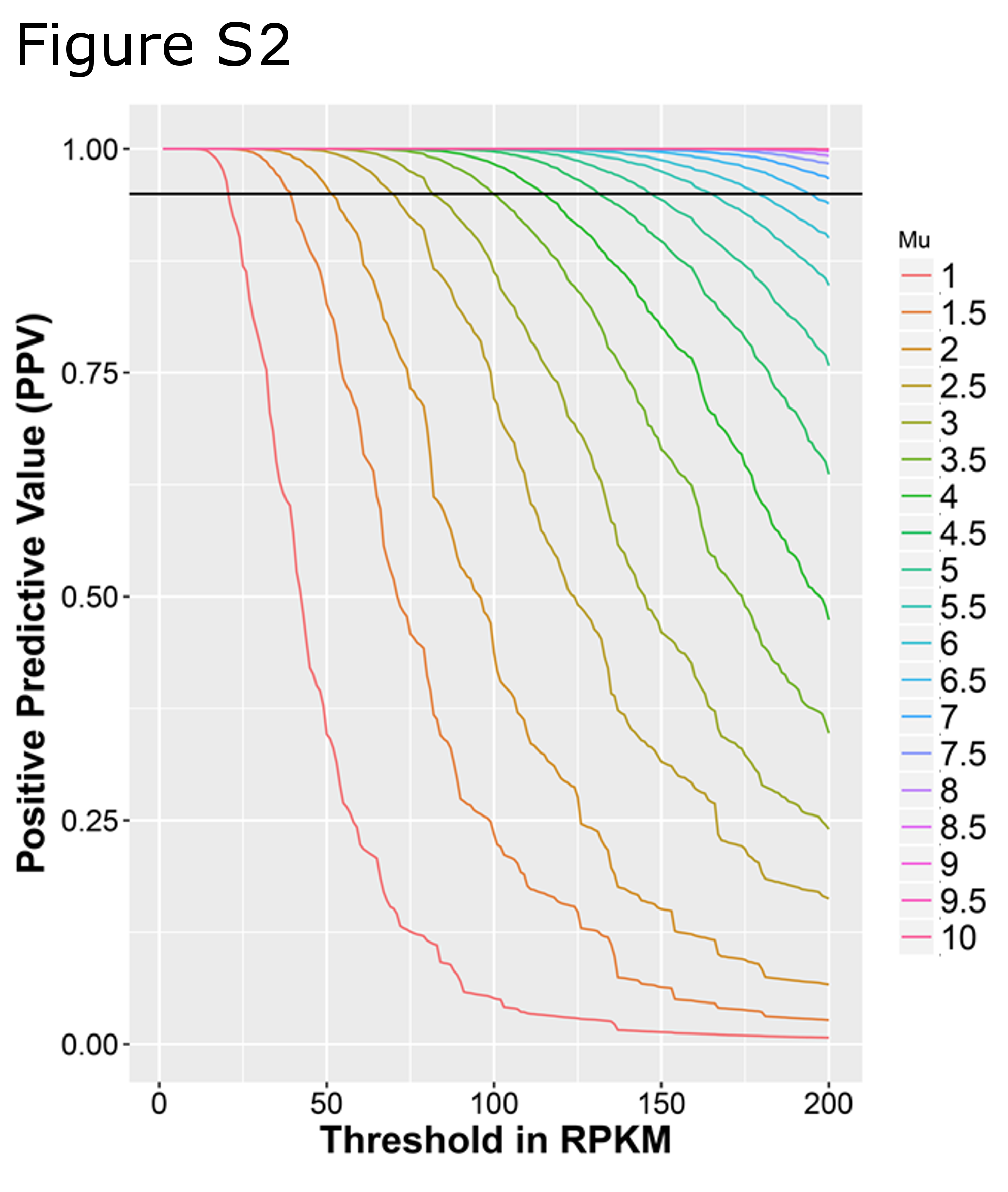

Supplement: Supplementary Data [file gky1223_supplemental_files.zip › Figure_S2.tiff]

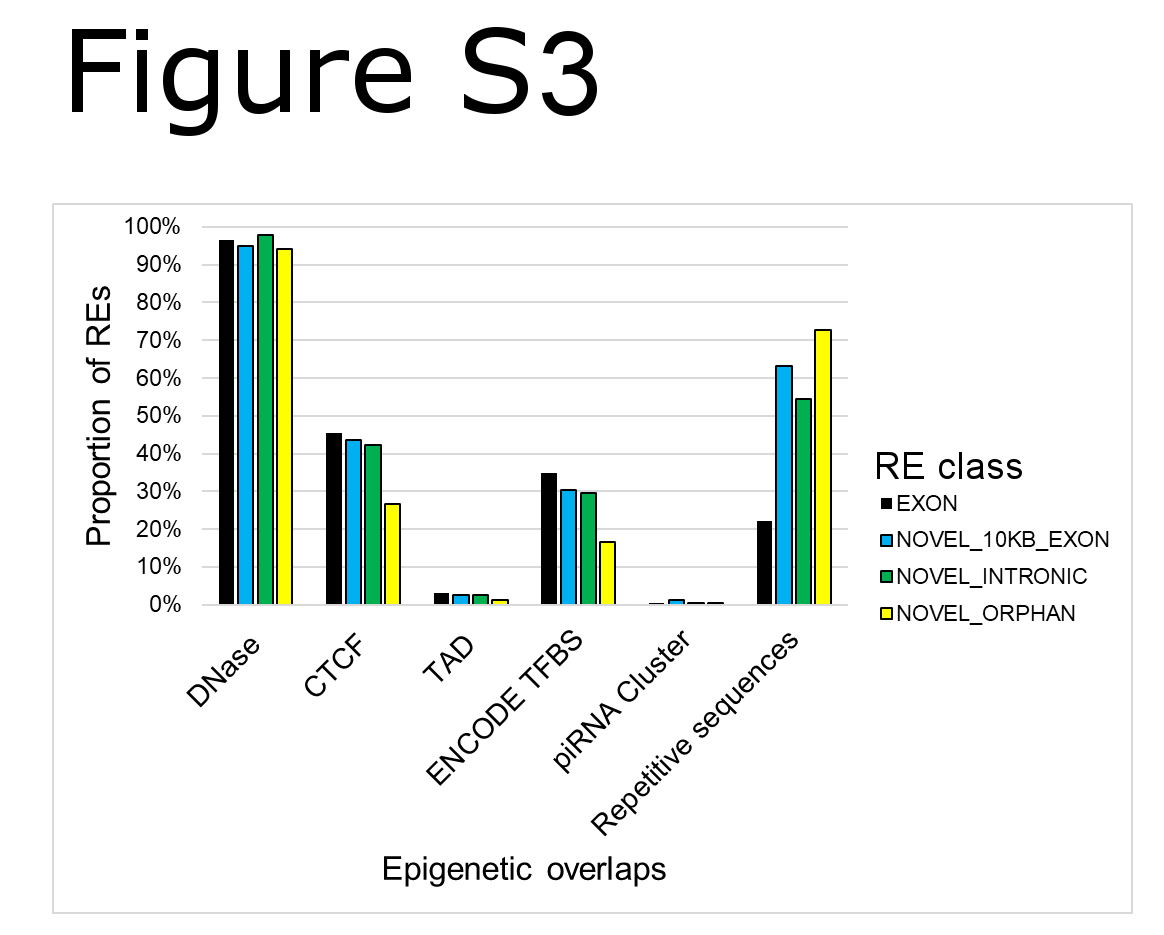

Supplement: Supplementary Data [file gky1223_supplemental_files.zip › Figure_S3.tiff]

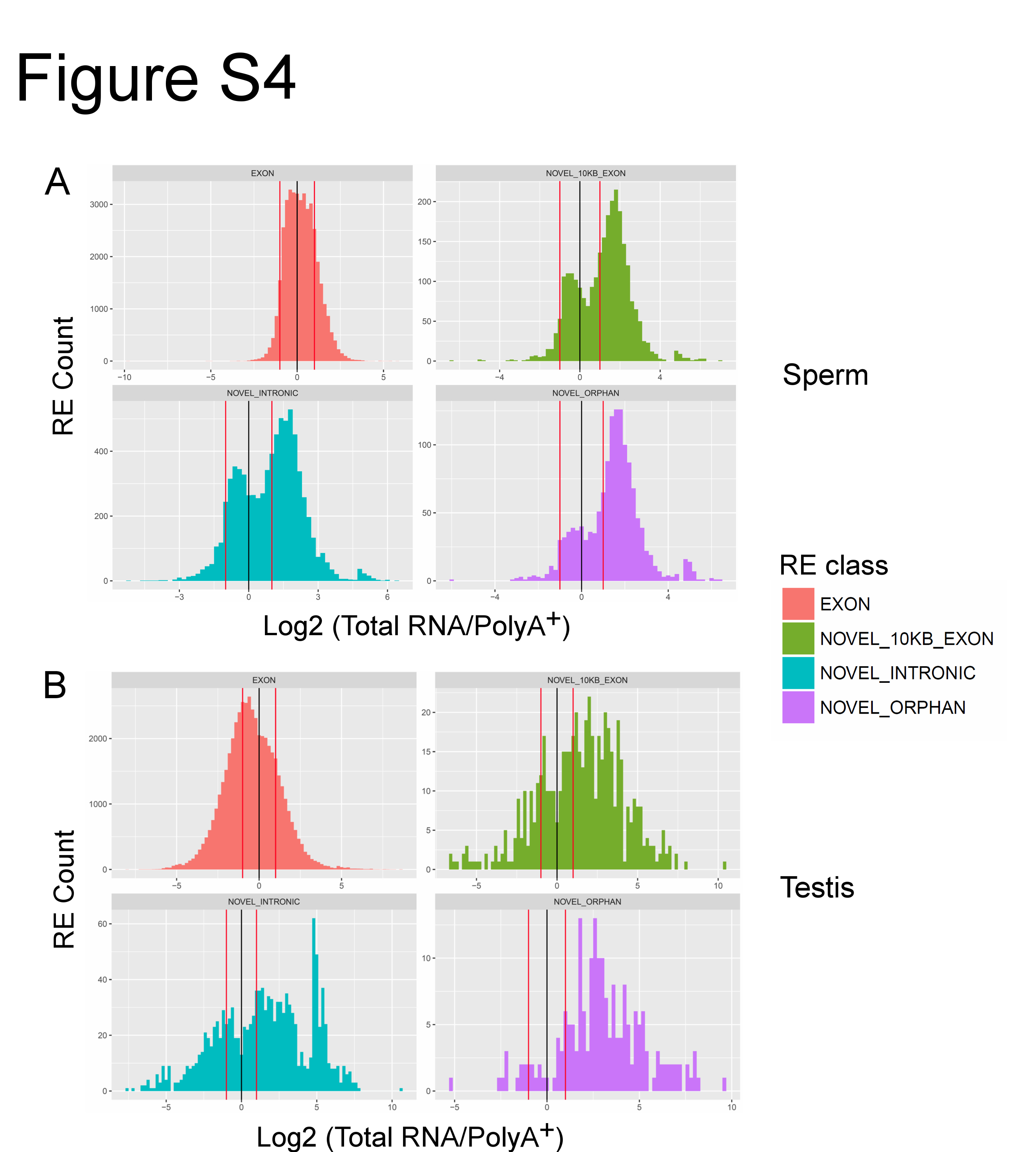

Supplement: Supplementary Data [file gky1223_supplemental_files.zip › Figure_S4.tiff]

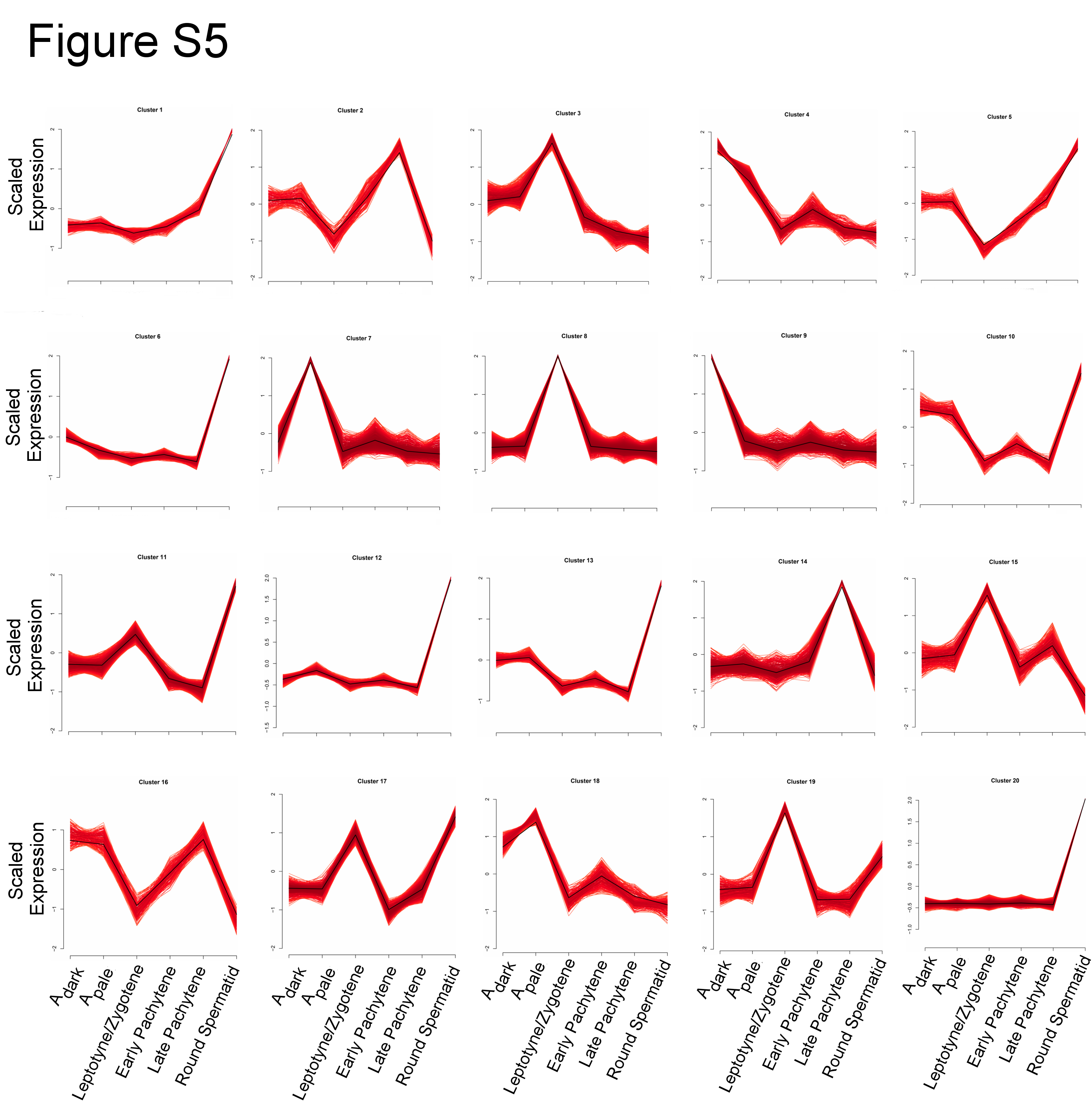

Supplement: Supplementary Data [file gky1223_supplemental_files.zip › Figure_S5.tiff]

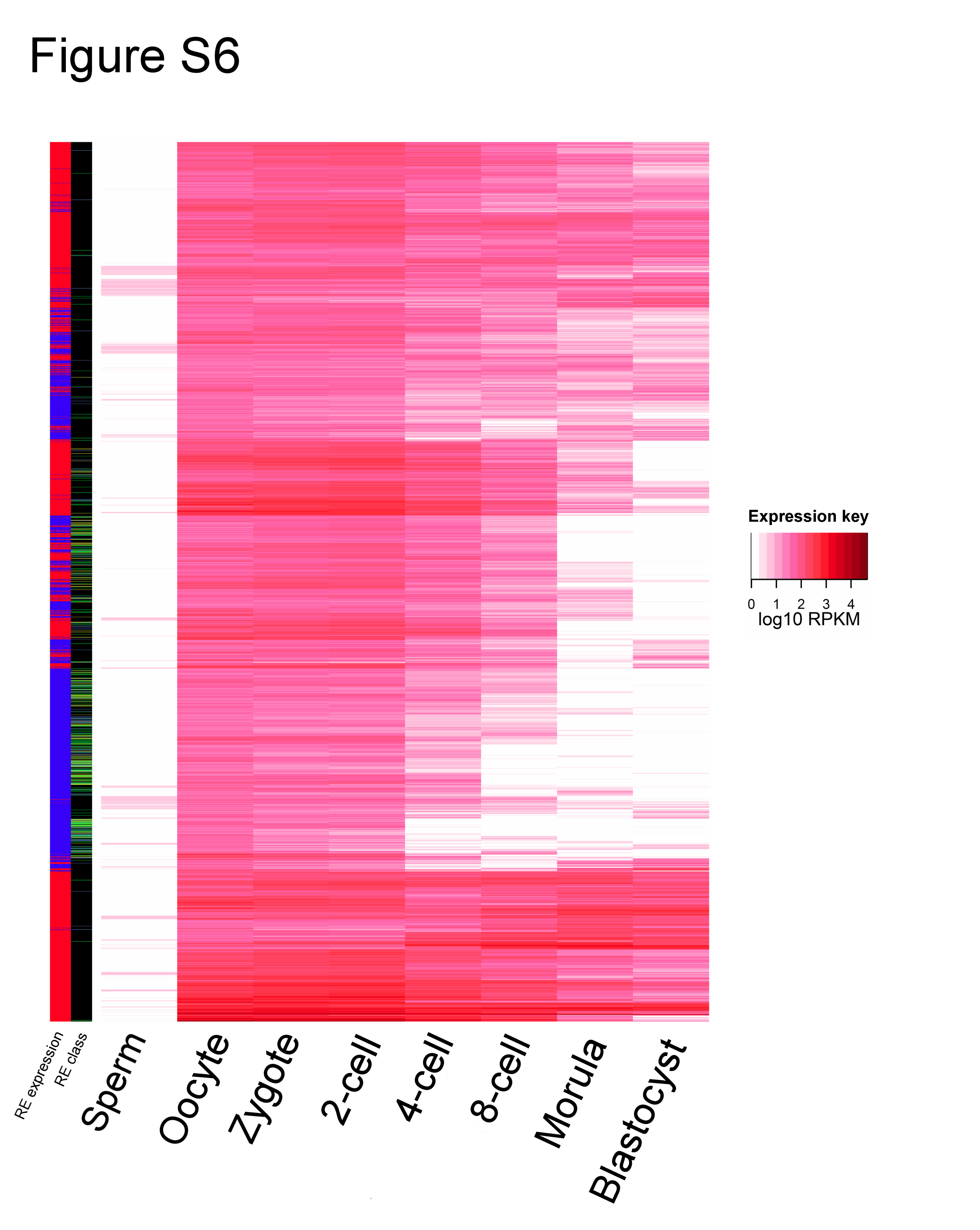

Supplement: Supplementary Data [file gky1223_supplemental_files.zip › Figure_S6.tiff]

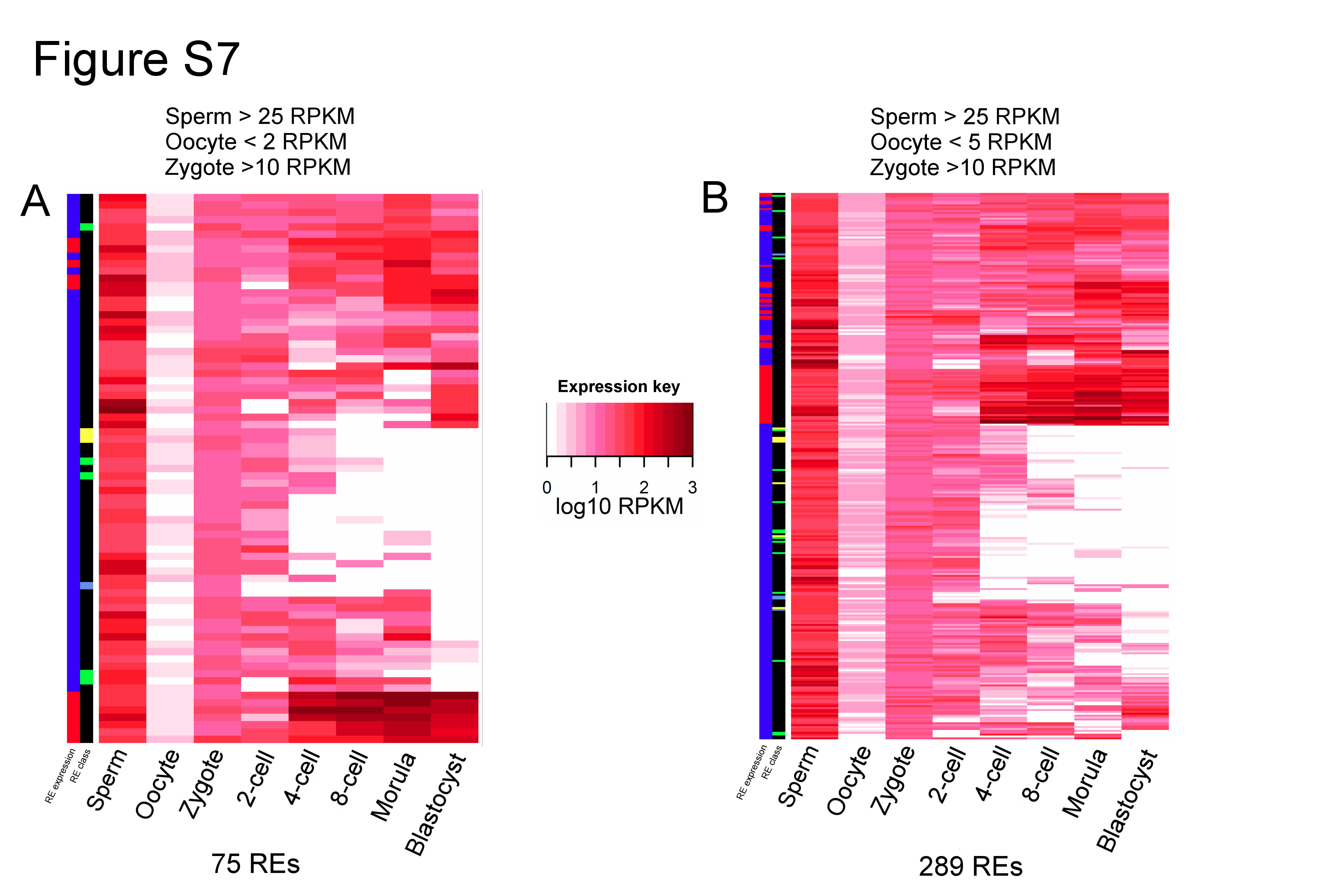

Supplement: Supplementary Data [file gky1223_supplemental_files.zip › Figure_S7.tiff]

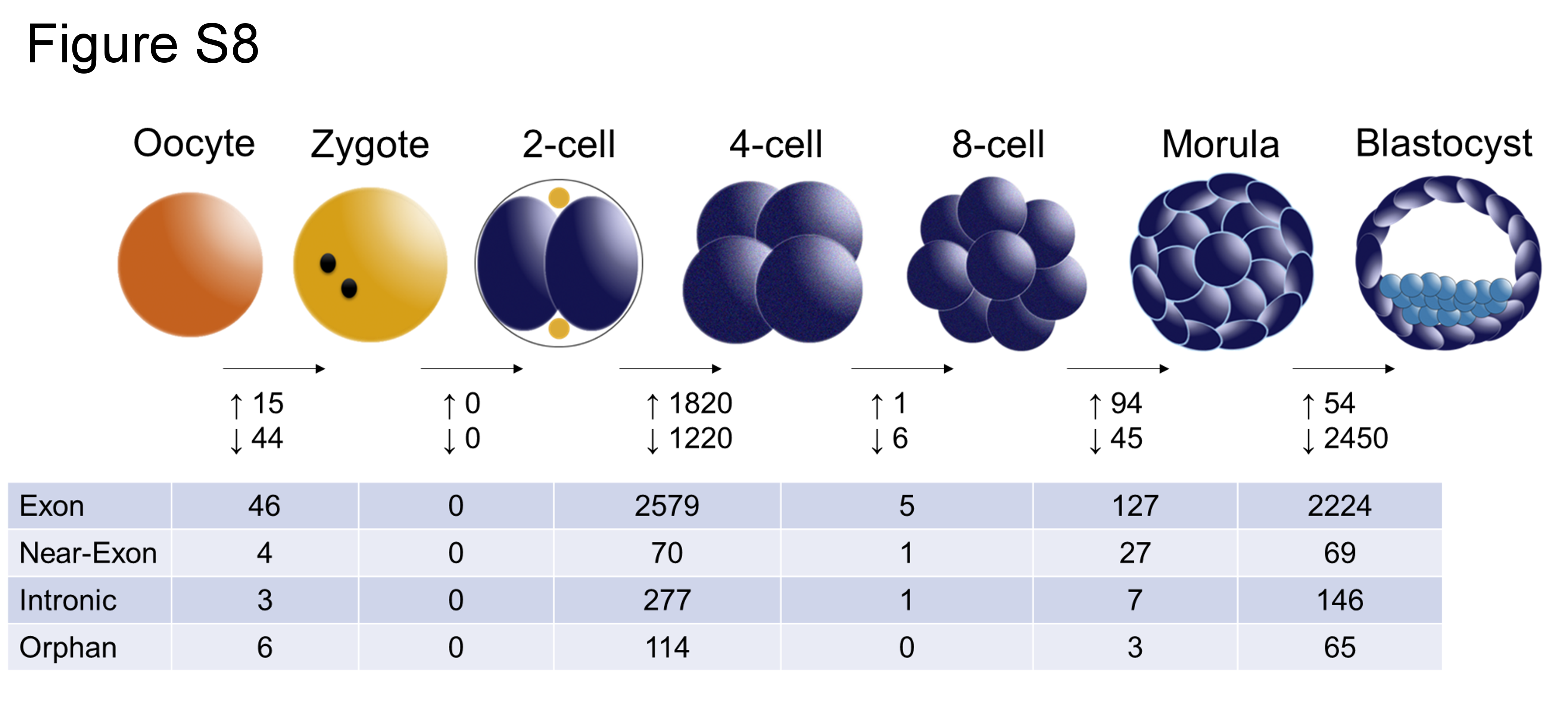

Supplement: Supplementary Data [file gky1223_supplemental_files.zip › Figure_S8.tiff]

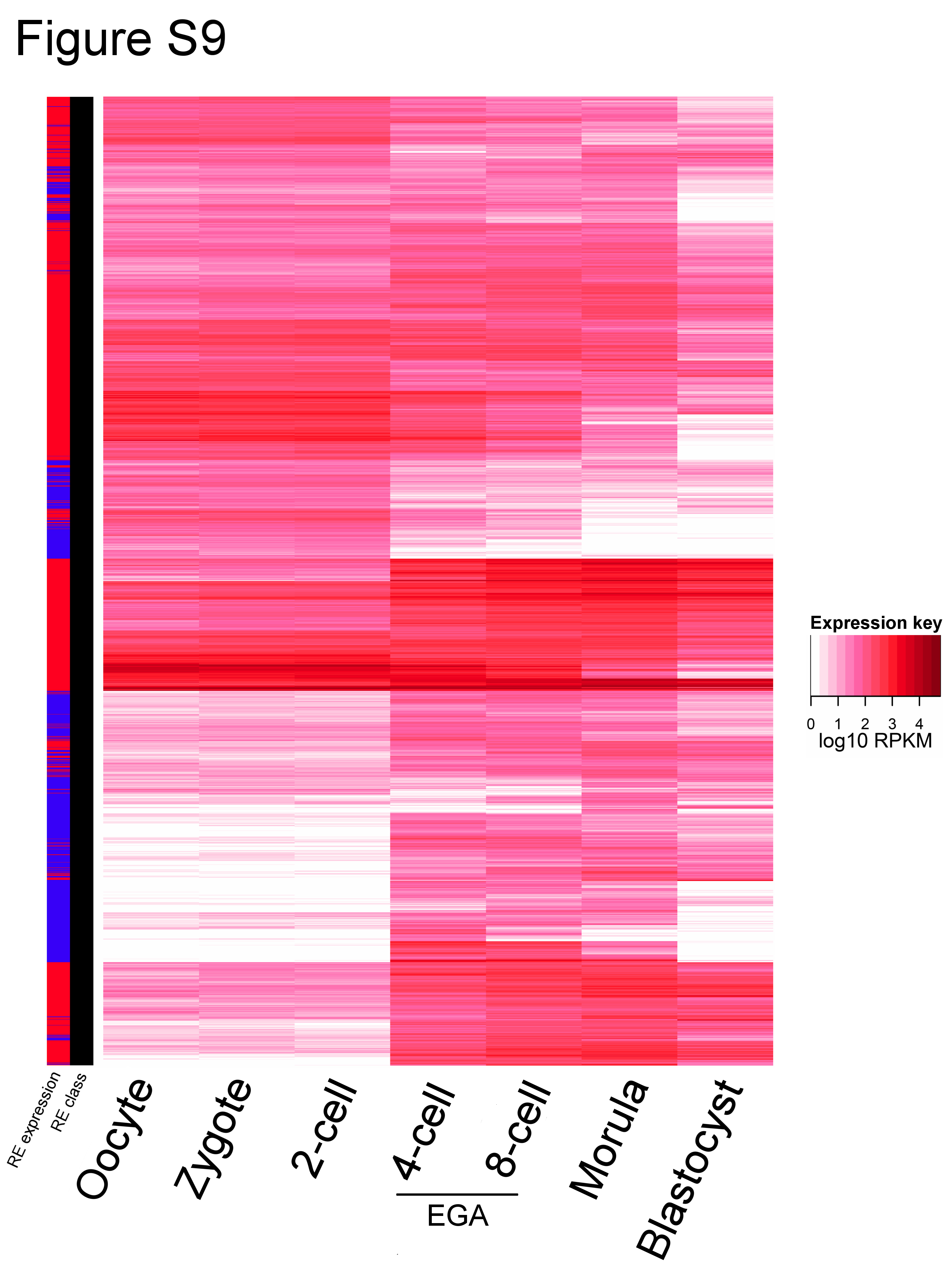

Supplement: Supplementary Data [file gky1223_supplemental_files.zip › Figure_S9.tiff]
